# Genetic and Functional Evidence Links Germline Biallelic Inactivating Variants in *WWOX* to Histological Mixed‐Type Thyroid Cancer

**DOI:** 10.1002/advs.202507602

**Published:** 2025-10-22

**Authors:** Xiaopeng Zhang, Jian Qi, Jialiang Wang, Zhipeng Wang, Yongguang Wang, Zongtao Hu, Ao Xu, Bo Hong, Hongzhi Wang

**Affiliations:** ^1^ Hefei Cancer Hospital of CAS Institute of Health and Medical Technology Hefei Institutes of Physical Science Chinese Academy of Sciences (CAS) Hefei Anhui 230031 China; ^2^ Science Island Branch Graduate School of the University of Science and Technology of China Hefei Anhui 230026 China; ^3^ Department of Pathology The First Affiliated Hospital of USTC Division of Life Sciences and Medicine University of Science and Technology of China (USTC) Hefei Anhui 230036 China

**Keywords:** DNA damage repair, germline variant, loss of function, lysosomal degradation, tumor suppressor, WWOX

## Abstract

Despite WWOX's established role as a tumor suppressor, conclusive evidence linking germline *WWOX* loss‐of‐function variants to oncogenesis remains scarce. Two germline homozygous *WWOX* missense variants (p.P252A and p.P282A) are identified in a patient with histological mixed‐type thyroid cancer. In vitro and in vivo functional assays demonstrate that both WWOX^P252A^ and WWOX^P282A^ mutants exhibit complete loss of tumor‐suppressive activity, failing to inhibit tumor cell growth and invasion. The WWOX^P252A^ mutant undergo accelerated degradation via HSC70 chaperone‐mediated autophagy in the lysosome. Furthermore, both P252A and P282A variants impair the WWOX protein's critical role in DNA damage repair. A nucleotide excision repair‐related protein, POLE4, is identified to interact with WWOX, but not with the WWOX^P282A^ mutant. Finally, low WWOX expression is found to be associated with epithelial‐mesenchymal transition and aggressive phenotype in thyroid cancer. These findings provide the first genetic and functional evidence that germline *WWOX* loss‐of‐function variants drive cancer pathogenesis by perturbing multiple tumor‐suppressive mechanisms.

## Graphical abstract

The role of germline WWOX loss‐of‐function variants in oncogenesis has remained poorly characterized. This study provides the first comprehensive genetic and functional evidence that such variants promote tumorigenesis through the disruption of multiple tumor‐suppressive mechanisms. These findings establish a role for WWOX in hereditary cancer predisposition and have clinical implications for risk stratification.

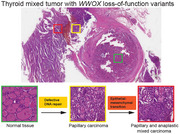

## 1 Introduction

The gene encoding *WWOX* (WW domain‐containing oxidoreductase), spanning chromosome region 16q23.1‐16q23.2 and crossing 1.1 million base pairs, ranks among the largest genes in the human genome.^[^ ^1^ ^]^ The *WWOX* gene is located within the FRA16D chromosomal region, a common fragile site characterized by susceptibility to chromosomal breakage, which consequently leads to frequent deletion events of the gene.^[^ ^2^ ^]^ The *WWOX* gene has been implicated in the pathogenesis of multiple cancer types, with recurrent deletions in diverse malignancies including bladder, colon, esophageal, ovarian, stomach, and uterine cancers.^[^ ^1^ ^]^ Mice harboring either homozygous or heterozygous deletion of *WWOX* exhibited spontaneous tumorigenesis, developing malignancies resembling human osteosarcoma, pulmonary, and mammary carcinomas.^[^ ^3^, ^4^ ^]^ These studies demonstrated that WWOX functions as a tumor suppressor gene. A hallmark feature of classical tumor suppressor genes such as *BRCA1*, *BRCA2*, and *TP53* is their propensity to acquire loss‐of‐function point mutations that disrupt their tumor‐suppressive activities.^[^ ^5^ ^]^ However, point mutations in the *WWOX* gene associated with functional loss of its tumor suppressor activity have rarely been identified to date. Therefore, this paucity of reported cancer‐associated loss‐of‐function variants positions WWOX as a non‐canonical tumor suppressor gene in current oncogenetic paradigms.^[^ ^6^, ^7^ ^]^

There has been accumulating evidence showing that downregulation of WWOX expression promotes tumor development in various cancer types.^[^ ^8^ ^]^ In breast and ovarian cancers, reduced WWOX expression drove metastatic progression by triggering the signaling pathways involving JNK/STAT3 activation, TGF‐β‐mediated SMAD phosphorylation, and Myc‐dependent transcriptional reprogramming, thereby facilitating coordinated epithelial‐mesenchymal transition (EMT) and extracellular matrix remodeling.^[^ ^9^, ^10^, ^11^, ^12^, ^13^ ^]^ Husanie et al. have demonstrated that *Wwox* conditional deletion in genetically‐engineered mice accelerated the formation of pancreatic cancer by enhanced macrophage infiltration and cancer stemness.^[^ ^14^ ^]^ In liver cancer, loss of WWOX leads to metabolic reprogramming by increased HIF1α level, thereby increasing cancer cell proliferation.^[^ ^15^, ^16^ ^]^ Furthermore, previous studies have demonstrated that *WWOX*‐deficient cells were more susceptible to genomic instability, due to their direct participation in DNA damage repair. WWOX was involved in the repair of DNA double‐strand breaks (DSB) and single‐strand breaks (SSB) via the interaction of ATM and ATR.^[^ ^17^, ^18^ ^]^ Schrock et al. indicated that WWOX interacted with BRCA1, and the loss of WWOX expression significantly altered DSB repair pathways, from the decreased non‐homologous end joining (NHEJ) to the enhanced homology‐directed repair (HDR) and single‐strand annealing (SSA).^[^ ^19^ ^]^ Thus, WWOX exhibits tumor suppressive activity by the inhibition of cellular proliferation and invasion, as well as the maintenance of genomic stability.

WWOX functional loss can occur through several distinct mechanisms, including genomic deletion, promoter hypermethylation, point mutations, or post‐translational modification, etc.^[^ ^20^, ^21^ ^]^ Genomic deletion is the main mechanism leading to the loss of WWOX expression.^[^ ^22^ ^]^ The promoter hypermethylation also silenced WWOX expression in breast, lung, and pancreatic cancers.^[^ ^23^, ^24^ ^]^ In prostate cancer, the Ack‐1 tyrosine kinase mediates phosphorylation of WWOX, triggering its polyubiquitination and subsequent proteasomal degradation.^[^ ^25^, ^26^ ^]^ Germline biallelic loss‐of‐function variants in the *WWOX* gene have been identified, but cause WWOX‐related epileptic encephalopathy (WOREE) syndrome, an autosomal recessive neurological disorder characterized by severe epileptic encephalopathy, developmental delay, and premature death.^[^ ^27^, ^28^ ^]^ The predominant *WWOX* variants related to WOREE syndrome were nonsense and frameshift variants, such as R54^*^, D16fs, W335^*^, and E306fs, etc. The missense variants (P47R, Q230P, and G137E) were also observed in WOREE syndrome individuals.^[^ ^27^ ^]^ Despite WWOX's well‐established role as a tumor suppressor, germline loss‐of‐function variants in this gene were rarely found in cancer patients. Our recent study identified a germline homozygous 5‐bp insertion variant (NM_016373: c.1425_1426insGTAAA) in the *WWOX* 3′‐UTR region, in a young proband presenting with endometrioid and clear cell adenocarcinoma mixed ovary cancer, and tubulo‐villous and mucinous adenocarcinoma mixed colon cancer.^[^ ^29^ ^]^ Case‐control association studies have revealed that a single nucleotide polymorphism (SNP) rs3764340 (*WWOX* p.P282A) was associated with a higher risk of cervical, thyroid, esophageal, and lung cancers.^[^ ^30^, ^31^, ^32^, ^33^ ^]^ However, the loss of tumor suppressor activity mediated by *WWOX* variants has not been functionally validated, leaving their oncological relevance uncertain.

In the study, by whole‐exome sequencing (WES), we identified two germline homozygous *WWOX* variants (p.P252A and p.P282A) in a young male presenting with a histological mixed‐type thyroid cancer (co‐existing papillary and anaplastic carcinoma). This study provides the first functional evidence demonstrating tumor suppressor inactivation of *WWOX* variants through in vitro and in vivo models, while mechanistically elucidating the molecular basis of WWOX functional impairment.

## 2 Results

### 2.1 Germline Homozygous Variants in the *WWOX* Gene are Identified in a Patient with a Thyroid Mixed Tumor of Papillary and Anaplastic Carcinoma

**Figure 1:**
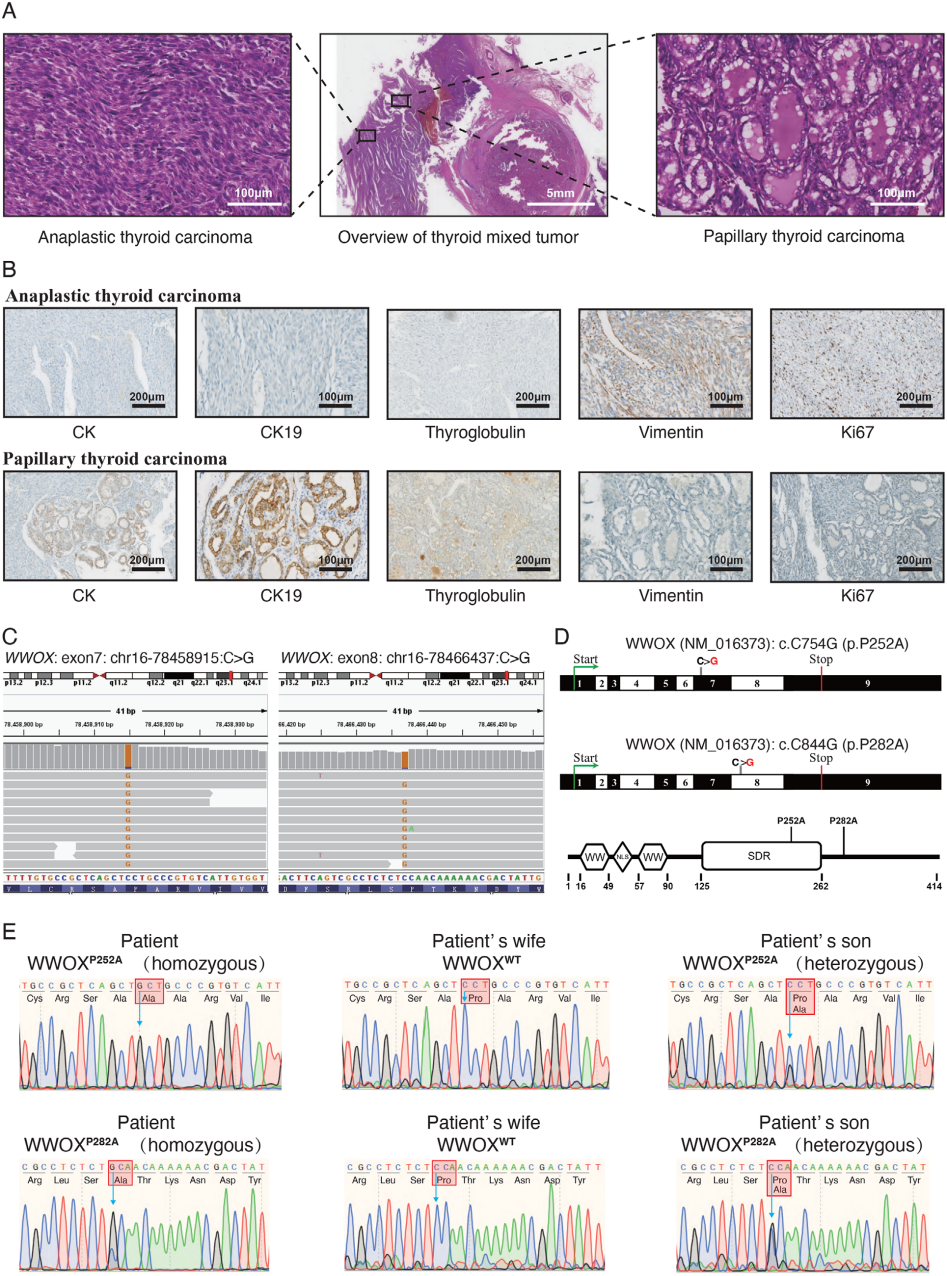
Germline homozygous *WWOX* P252A and P282A variants are identified in a patient with thyroid mixed tumor of papillary and anaplastic carcinoma. A) H&E staining analysis of thyroid mixed tumor (middle panel) of the patient. The thyroid mixed tumor includes two histological types, anaplastic carcinoma (right panel) and papillary carcinoma (left panel), which are enlarged. B) Representative images of IHC staining for the two histological types, anaplastic carcinoma and papillary carcinoma in the thyroid mixed tumor. CK (Cytokeratin) and CK19 (Cytokeratin 19) are markers of epithelial cells. Thyroglobulin is the maker of differentiated degree of thyroid cells. Vimentin is the marker of mesenchymal cells. Ki‐67 is the marker of proliferation. C) IGV (integrative genomics viewer) visualization of the homozygous *WWOX* germline variants detected in adjacent normal tissue of the thyroid mixed tumor by WES. One variant is located on exon 7 in the *WWOX* gene (WWOX^P252A^, left panel). The other variant is located on exon 8 in the *WWOX* gene (WWOX^P282A^, right panel). D) Schematic diagram for the locations of P252A and P282A variants in the *WWOX* transcript (top panel) and WWOX protein (bottom panel). The translation start codon is shown in green. The stop codon is shown in red. The P252A and P282A missense variants are shown in grey. E) Validation of the two germline homozygous *WWOX* variants (WWOX^P252A^ and WWOX^P282A^) in the patient by Sanger sequencing. The two germline *WWOX* variants are homozygous in the patient (left panel), heterozygous in the patient's son (right panel), and wild type in the patient's wife (middle panel).

A 35‐year‐old Chinese male patient was admitted to The First Affiliated Hospital of USTC (University of Science and Technology of China) on November 6, 2015, presenting with a large mass in the right neck region (Figure S1, Supporting Information). After surgery, the large tumor (8 cm×6 cm×4.5 cm) was diagnosed by pathological examinations. H&E staining indicated that the tumor was composed predominantly of spindle cells, which were diagnosed as anaplastic thyroid carcinoma (**Figure** **1**A). Besides this, the component of papillary thyroid carcinoma was observed in the tumor (Figure 1A). Immunohistochemical (IHC) analysis showed that the anaplastic thyroid carcinoma expressed vimentin, while it lacked the expression of CK, CK19, and thyroglobulin. On the contrary, the papillary thyroid carcinoma stained positive for CK, CK19, and thyroglobulin, and negative for vimentin. The staining of proliferation marker Ki‐67 was stronger in the anaplastic thyroid carcinoma, compared with the papillary thyroid carcinoma (Figure 1B). Finally, the tumor in the right neck region of the patient was diagnosed as a thyroid mixed tumor of papillary and anaplastic carcinoma.

The thyroid mixed tumor of papillary and anaplastic carcinoma is very malignant and rare. The patient passed away two years after diagnosis. Since the patient was very young, we considered that the hereditary factor could be related to the highly aggressive malignant phenotype. Therefore, the patient's tumor tissue and adjacent normal tissue were sequenced by whole‐exome sequencing (WES). The paired normal tissue was subjected to WES with an average sequencing depth of 35× (Table S1, Supporting Information). The WES identified seven germline damaging variants that were predicted to impact protein function by all algorithms, including SIFT, Polyphen, MutationAssessor, MutationTaster, LRT, and FATHMM (Table S2, Supporting Information). The other deleterious variants, including frameshift and nonsense variants, were listed in Table S3 (Supporting Information). The missense P252A variant in the *WWOX* gene was selected for further analysis based on its homozygosity and previously reported tumor‐suppressive function. Integrative Genomics Viewer (IGV) displayed that the variant was located on exon 7 in the *WWOX* gene, resulting in a homozygous C‐to‐G missense variant (NM_016373:c.C754G:p.P252A). Besides the P252A variant, WES identified the other homozygous variant in the *WWOX* gene that was located on exon 8, resulting in a C‐to‐G missense variant (NM_016373:c.C844G:p.P282A) (Figure 1C,D; Table S4, Supporting Information). By Sanger sequencing, we further confirmed the homozygosity of the two *WWOX* germline variants using the genomic DNA from the adjacent muscle normal tissue of the patient's tumor. In addition, the son of the patient harbored heterozygous alleles and had no cancer phenotype. The wife of the patient harbored wild‐type alleles (Figure 1E). In the patient's tumor sample, somatic mutations were also identified by WES with an average sequencing depth of 135× (Table S1, Supporting Information). Somatically mutated cancer‐driver genes included *MSH6*, *ARID1B*, *ATP2A1*, *TP53* and *BTK* (Table S5, Supporting Information). In summary, germline homozygous P252A and P282A variants in *WWOX* were identified in a patient with a rare and malignant thyroid tumor composed of mixed papillary and anaplastic carcinoma.

### 2.2 The WWOX^P252A^ and WWOX^P282A^ Mutants Lose the Function of the Tumor Suppressor

**Figure 2:**
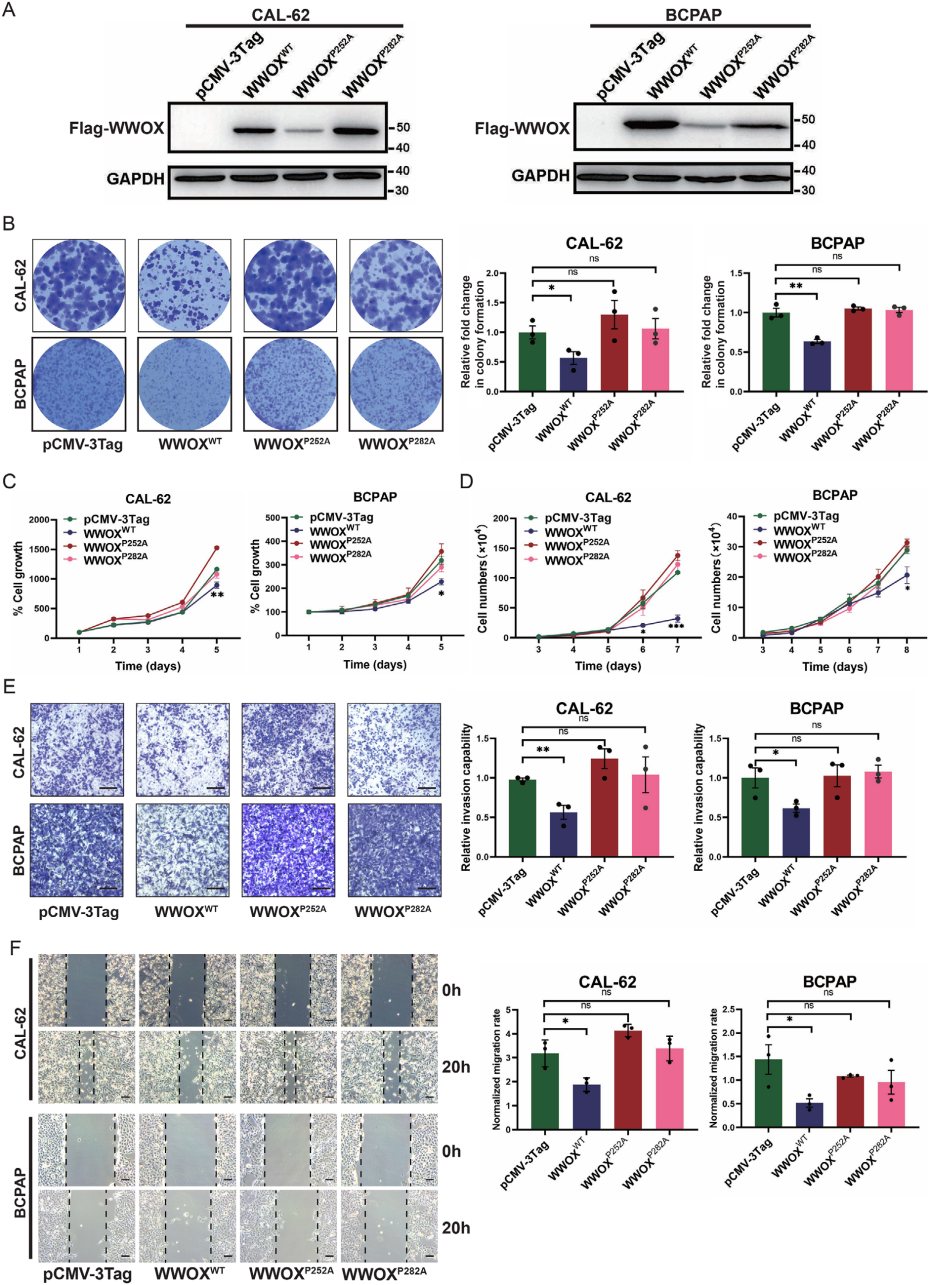
The WWOX^P252A^ and WWOX^P282A^ mutants lose the function of a tumor suppressor in vitro. A) Western blot validates the stable over‐expression of Flag‐tagged wild‐type (WT), P252A, and P282A mutant WWOX proteins in CAL‐62 and BCPAP thyroid cancer cells. Expression of the Flag‐tagged WWOX proteins (Flag‐WWOX) was detected using an anti‐FLAG antibody. B) Colony formation assay indicates that the ability of colony formation is significantly reduced in CAL‐62 and BCPAP cells with stable over‐expression of wild‐type WWOX protein, but not in cells with stable over‐expression of WWOX^P252A^ and WWOX^P282A^ mutants. The colonies were quantified relative to the empty vector and shown by mean ± SEM from three independent experiments (*n* = 3, Student's t‐test). C) The CellTiter‐Glo Luminescent assay shows that stable over‐expression of wild‐type WWOX significantly inhibits the proliferation of CAL‐62 and BCPAP cells, whereas over‐expression of WWOX^P252A^ and WWOX^P282A^ mutants does not exert such an inhibitory effect. Error bars represent mean ± SEM (*n* = 3, Student's t‐test). D) Cell counting shows that the growth of CAL‐62 and BCPAP cells is significantly inhibited by stable over‐expression of wild‐type WWOX, but not by stable over‐expression of WWOX^P252A^ and WWOX^P282A^ mutants. Error bars represent mean ± SEM (*n* = 3, Student's t‐test). E) Matri‐gel invasion assay indicates that stable over‐expression of wild‐type WWOX significantly inhibits the invasion of CAL‐62 and BCPAP cells, but WWOX^P252A^ and WWOX^P282A^ mutants lose the inhibitory ability of cell invasion. The invaded cells were quantified and shown by mean ± SEM from three independent experiments (*n* = 3, Student's t‐test). F) Wound‐healing assay indicates that stable over‐expression of wild‐type WWOX significantly inhibits the migration of CAL‐62 and BCPAP cells, whereas WWOX^P252A^ and WWOX^P282A^ mutants lose the inhibitory ability of cell migration. Wound closure at 20 h was normalized to the initial wound area at 0 h, and quantified as a migration rate. The data is shown by mean ± SEM from three independent experiments (*n* = 3, Student's t‐test).

To determine the effect of the two missense variants on WWOX protein function, we established two thyroid cancer cell lines (CAL‐62 and BCPAP) with the stable overexpression of wild‐type, P252A, or P282A mutated WWOX protein (**Figure** **2**A). To investigate the impact of WWOX mutants on cell growth, a colony formation assay was performed. The results showed that the number of colonies was markedly decreased in both thyroid cancer cells stably overexpressing wild‐type WWOX (Figure 2B). However, when WWOX^P252A^ or WWOX^P282A^ mutant was stably expressed in CAL‐62 and BCPAP thyroid cancer cells, the two mutants were not able to inhibit colony formation (Figure 2B). Similarly, cell viability assay indicated that the over‐expression of wild‐type WWOX inhibited the growth of thyroid cancer cells, but WWOX^P252A^ or WWOX^P282A^ mutants lost the ability to inhibit growth (Figure 2C). Cell counting further indicated that the WWOX^P252A^ or WWOX^P282A^ mutant had no function of growth inhibition (Figure 2D). Moreover, we examined the effect of WWOX^P252A^ and WWOX^P282A^ mutants on cell invasion. The results demonstrated that cell invasion was reduced in CAL‐62 and BCPAP cells stably expressing wild‐type WWOX, compared with the cells stably expressing pCMV‐3Tag empty vector (Figure 2E). But the inhibition of cell invasion was not observed in CAL‐62 and BCPAP cells stably overexpressing WWOX^P252A^ or WWOX^P282A^ mutant (Figure 2E). Likewise, wound healing assay revealed that the over‐expression of wild‐type WWOX was able to inhibit cell migration significantly in both thyroid cancer cells, but this inhibitory effect was not observed in the WWOX^P252A^ or WWOX^P282A^ mutant cells (Figure 2F). Given that the patient with a thyroid mixed tumor harbored both the *WWOX* P252A and P282A variants, we further evaluated the functional consequences of this double mutation on WWOX protein. We generated CAL‐62 and BCPAP cell lines stably overexpressing the WWOX double mutant (P252A/P282A) and subsequently assessed their growth and invasive capabilities. Similar to the WWOX^P252A^ and WWOX^P282A^ single mutants, the WWOX^P252A/P282A^ double mutant failed to inhibit the growth and invasion of CAL‐62 and BCPAP cells (Figure S2, Supporting Information).

**Figure 3:**
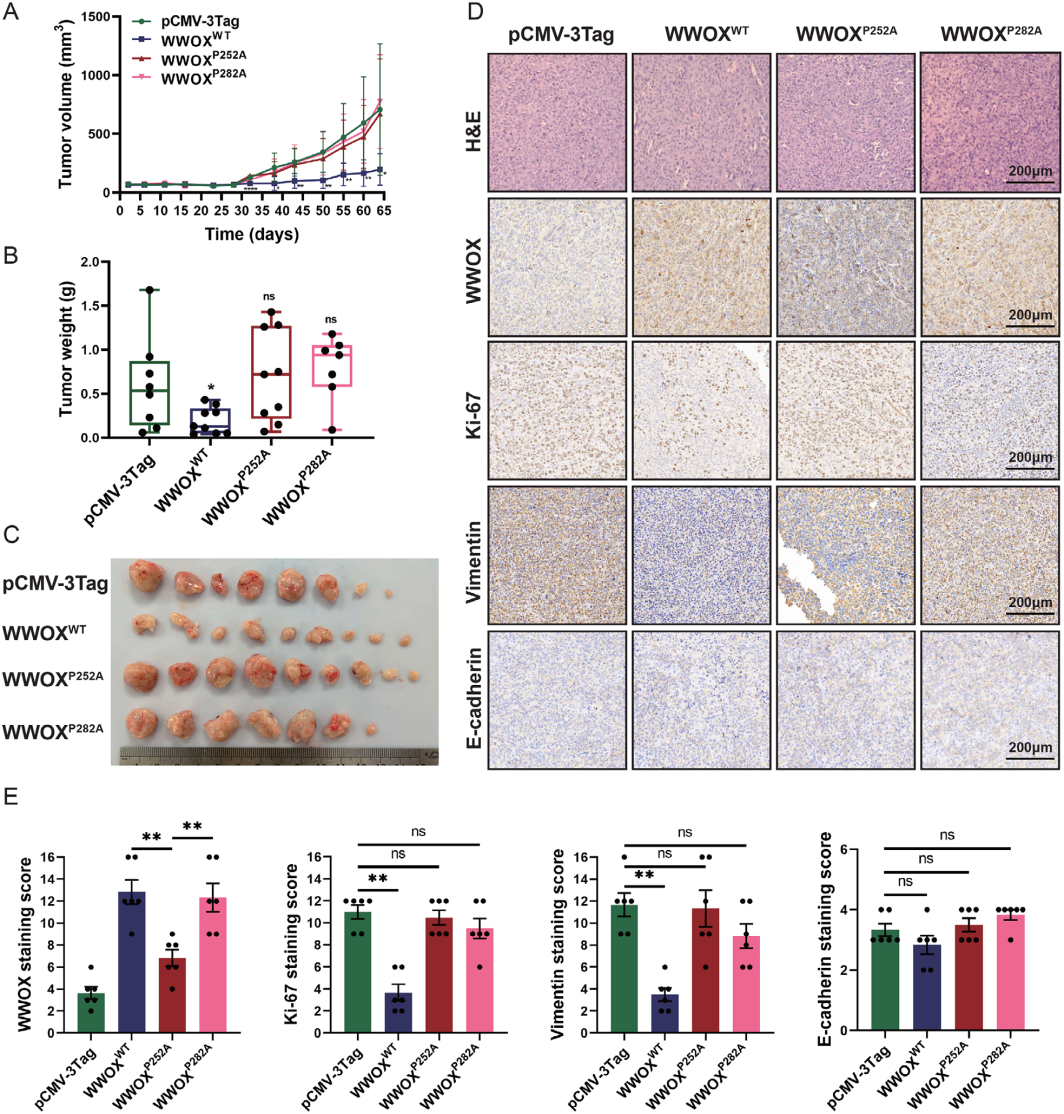
The WWOX^P252A^ and WWOX^P282A^ mutants lose the function of a tumor suppressor in vivo. A) Subcutaneous tumorigenesis assay in nude mice indicates that wild‐type WWOX, but not WWOX^P252A^ and WWOX^P282A^ mutants, inhibits the growth of thyroid cancer xenografts. CAL‐62 cells with stable overexpression of empty vector, wild‐type WWOX, WWOX^P252A^, and WWOX^P282A^ mutants were subcutaneously injected into nude mice. The tumor volume curve over a period of 9 weeks is shown by mean ± SEM (Student's t‐test). B) Tumor weights from different groups at the end of the experiment are shown by mean ± SEM (Student's t‐test). C) Images of tumors from different groups at the end of the experiment. D) H&E and IHC staining analysis of tumor tissues from different groups. E) The quantitative analysis for IHC staining of tumor tissues from different groups (*n* = 6, Mann‐Whitney U test).

Finally, we established xenograft models in mice subcutaneously inoculated with CAL‐62 cells stably expressing pCMV‐3Tag empty vector, WWOX^WT^, WWOX^P252A,^ or WWOX^P282A^ mutant. The results indicated that over‐expression of wild‐type WWOX significantly suppressed tumor growth in vivo, compared with the group of empty vector (**Figure** **3**A). However, the WWOX^P252A^ or WWOX^P282A^ mutant did not have the inhibitory effect on tumor growth (Figure 3A). At the end of the in vivo experiment, we dissected the tumors from the mice. The tumors’ weight from the wild‐type WWOX group was significantly reduced, compared with the empty vector group. The WWOX^P252A^ and WWOX^P282A^ mutants did not appear to affect the weight of the tumors, indicating the loss of tumor suppressor function in vivo (Figure 3B,C). IHC staining showed that the expression of Ki‐67 (proliferation marker) and vimentin (mesenchymal marker) was significantly reduced in CAL‐62 xenografts stably expressed with wild‐type WWOX, compared with CAL‐62 xenografts stably expressed with empty vector, WWOX^P252A,^ or WWOX^P282A^ mutant (Figure 3D,E; Figure S3, Supporting Information). In summary, these results indicate that WWOX^P252A^ and WWOX^P282A^ mutants lose the inhibitory ability of cell proliferation and invasion in vitro and in vivo.

### 2.3 WWOX^P252A^ Mutant Protein is Degraded Quickly by Chaperone‐Mediated Autophagy in the Lysosome

**Figure 4:**
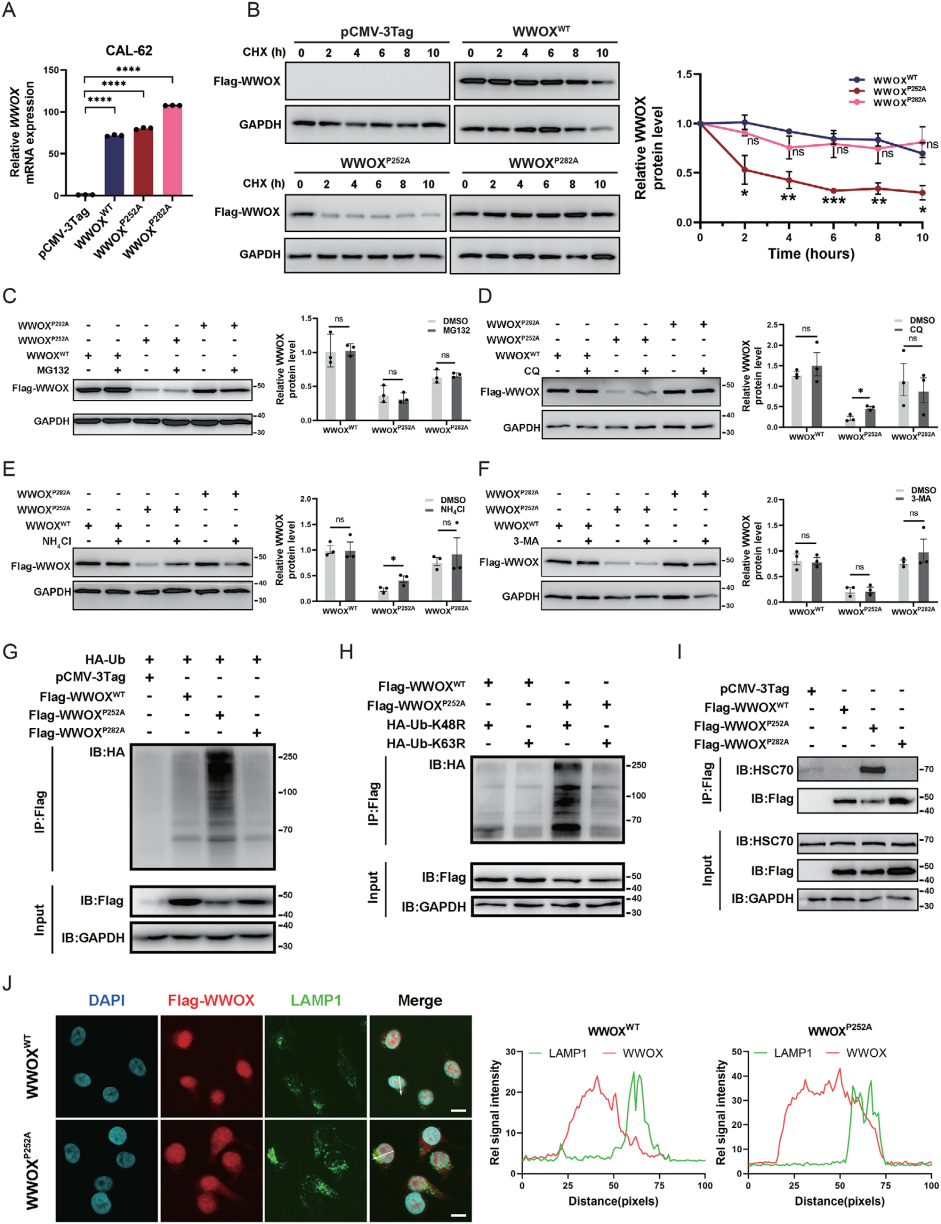
WWOX^P252A^ mutant protein undergoes rapid degradation via chaperone‐mediated autophagy in the lysosome. A) qRT‐PCR shows that WWOX mRNA level is dramatically elevated in CAL‐62 cells with stable over‐expression of wild‐type WWOX, WWOX^P252A^, and WWOX^P282A^ mutants. B) Cycloheximide (CHX) chase assay indicates the rapid degradation of WWOX^P252A^ mutant protein. CAL‐62 cells with stable over‐expression of empty vector, wild‐type, and mutant WWOX proteins were treated with 50 µg mL^−1^ CHX for the indicated time. The expression of Flag‐WWOX was detected by western blot, quantified by ImageJ, and then normalized to GAPDH loading control. The quantitative analysis is shown by mean ± SEM from three independent experiments (*n* = 3, Student's t‐test). C) Treatment of proteasome inhibitor MG‐132 (20 µm, 6 h) does not lead to accumulation of WWOX^WT^, WWOX^P252A,^ and WWOX^P282A^ proteins in CAL‐62 cells stably over‐expressing these constructs. The quantitative analysis for western blot is shown by mean ± SEM from three independent experiments (*n* = 3, Student's t‐test). D) Treatment of CAL‐62 cells stably overexpressing wild‐type or mutant WWOX proteins with the lysosome inhibitor chloroquine (CQ; 40 µm, 24 h) induces accumulation of WWOX^P252A^ mutant protein. The quantitative analysis for western blot is shown by mean ± SEM from three independent experiments (*n* = 3, Student's t‐test). E) Treatment of CAL‐62 cells stably overexpressing wild‐type or mutant WWOX proteins with the lysosome inhibitor NH_4_Cl (250 µm, 24 h) induces accumulation of WWOX^P252A^ mutant protein. The quantitative analysis for western blot is shown by mean ± SEM from three independent experiments (*n* = 3, Student's t‐test). F) Treatment with the autophagosome inhibitor 3‐MA (10 mm, 12 h) does not alter the expression levels of WWOX^WT^, WWOX^P252A,^ and WWOX^P282A^ proteins in CAL‐62 cells stably overexpressing these constructs. The quantitative analysis for western blot is shown by mean ± SEM from three independent experiments (*n* = 3, Student's t‐test). G) Co‐IP analysis indicates the high ubiquitination level of the WWOX^P252A^ mutant protein. HEK293T cells were transiently co‐transfected with Flag‐tagged wild‐type (WWOX^WT^) or mutant WWOX (WWOX^P252A^ or WWOX^P282A^) and HA‐tagged ubiquitin plasmids. Immunoprecipitation (IP) with anti‐FLAG antibody followed by immunoblotting (IB) with anti‐HA (ubiquitin) was performed. H) Co‐IP analysis indicates that the WWOX^P252A^ mutant protein is conjugated with a K63‐linked polyubiquitylation chain. HEK293T cells were co‐transfected with Flag‐tagged wild‐type (WWOX^WT^) or mutant WWOX (WWOX^P252A^) and HA‐Ub‐K48R (ubiquitin K48R mutant) or HA‐Ub‐K63R (ubiquitin K63R mutant) plasmids. Immunoprecipitation (IP) with anti‐FLAG antibody, followed by immunoblotting (IB) with anti‐HA (ubiquitin), was performed. Ub‐K63R reduces WWOX polyubiquitination of WWOX^P252A^ protein, compared to Ub‐K48R. I) Co‐IP analysis of protein interaction between WWOX and HSC70. Cell lysates of CAL‐62 cells with stable over‐expression of Flag‐tagged wild‐type (WWOX^WT^) or mutant WWOX (WWOX^P252A^ or WWOX^P282A^) were immunoprecipitated with anti‐FLAG antibody, followed by western blot with anti‐HSC70 antibody. J) Immunofluorescence shows that the WWOX^P252A^ mutant protein has a trend to be transferred to lysosomes. Immunofluorescence was performed in CAL‐62 cells with stable overexpression of Flag‐tagged WWOX^WT^ or WWOX^P252A^ mutant WWOX using anti‐FLAG (red) and anti‐LAMP1 (green, lysosome marker). Nuclei were stained using DAPI. The line graph indicates the signal intensity of each protein along the arrow bars (right panel). Scale bar: 10 µm.

As shown in Figures 2A and 3D, when wild‐type WWOX and the two mutated WWOX proteins were stably overexpressed in CAL‐62 and BCPAP cells, western blot and IHC analyses showed that the protein expression of WWOX^P252A^ mutant was much lower than the expression of wild‐type WWOX and WWOX^P282A^ mutant proteins. We further evaluated WWOX mRNA levels following the overexpression of wild‐type and mutated WWOX. In CAL‐62 cells over‐expressing WWOX^WT^, WWOX^P252A,^ and WWOX^P282A^, WWOX mRNA levels showed a remarkable induction compared with empty vector‐transfected cells, while the mRNA level of WWOX^P252A^ mutant was not decreased (**Figure** **4**A). Thus, we hypothesized that the reduced expression of the WWOX^P252A^ mutant resulted from accelerated turnover rather than an impairment in transcription.

First, we examined the turnover of the wild‐type and two mutated WWOX proteins. The stability of WWOX proteins was evaluated in CAL‐62 cells with stable overexpression of wild‐type WWOX and the two mutants in the presence of cycloheximide (CHX), a protein synthesis inhibitor. The results demonstrated that the WWOX^P252A^ mutant exhibited significantly enhanced protein degradation, compared to its wild‐type counterpart and the WWOX^P282A^ mutant (Figure 4B). Next, we treated cells with inhibitors of two major proteolytic pathways: proteasome (MG‐132) and lysosome (chloroquine, NH_4_Cl, and 3‐methyladenine). We found that the proteasome inhibitor, MG‐132 treatment, did not induce the accumulation of WWOX^WT^, WWOX^P252A^, and WWOX^P282A^ proteins (Figure 4C). Importantly, lysosome inhibitors, chloroquine (CQ) and NH_4_Cl treatment, restored WWOX^P252A^ protein level (Figure 4D,E), whereas no significant effect was observed upon treatment with autophagosome inhibitor 3‐methyladenine (3‐MA) (Figure 4F). Furthermore, CQ prevented the degradation of WWOX^P252A^ mutant protein in the presence of CHX (Figure S4, Supporting Information). These results suggest that the P252A mutation causes rapid degradation of WWOX protein through a lysosome‐dependent pathway.

Subsequently, we examined the ubiquitination level of wild‐type WWOX and the two mutated WWOX proteins. HEK293T cells were transiently transfected with HA‐Ub and FLAG‐WWOX (wild‐type, P252A, and P282A mutants). The level of polyubiquitylation chain was stronger in the WWOX^P252A^ mutant protein, compared to wild‐type and WWOX^P282A^ mutant proteins (Figure 4G). By analyzing the polyubiquitylation chain type of the WWOX^P252A^ mutant protein, we found that the K63‐linked ubiquitin was the predominant form. As shown in Figure 4H and Figure S5 (Supporting Information), a K63R mutant type of ubiquitin reduced the polyubiquitination of the WWOX^P252A^ mutant protein.

Autophagy is divided into three types: macroautophagy, microautophagy, and chaperone‐mediated autophagy.^[^ ^34^ ^]^ As shown in Figure 4F, the autophagosome inhibitor (3‐MA) showed no effects on the level of WWOX^P252A^ mutant protein. In chaperone‐mediated autophagy (CMA), cytosolic chaperone HSC70 recognizes substrate proteins by binding to their KFERQ‐like motif, leading to their translocation into the lysosome for degradation.^[^ ^35^ ^]^ Since the KFERQ‐like motif (LRSVQ at 187‐191 amino acids) exists on WWOX, we speculated that lysosomal degradation of WWOX^252A^ mutant protein occurred through chaperone‐mediated autophagy. As shown in Figure 4I, co‐immunoprecipitation (co‐IP) revealed an interaction between HSC70 and WWOX^P252A^ mutant protein, but not wild‐type WWOX protein and WWOX^P282A^ mutant protein. Furthermore, we investigated whether the WWOX^P252A^ mutant protein was taken to lysosomes. Immunofluorescence analysis indicated that the WWOX^P252A^ mutant protein was transferred to lysosomes, displaying co‐localization with lysosomal marker LAMP1 (Figure 4J).

Therefore, these results indicate that the WWOX^P252A^ mutant protein is not stable with accelerated degradation through chaperone‐mediated autophagy in the lysosome.

### 2.4 The Ability of DNA Damage Repair of WWOX^P252A^ and WWOX^P282A^ Mutants is Impaired

Previous studies have demonstrated that WWOX is involved in the process of DNA damage repair to maintain genomic stability.^[^ ^36^ ^]^ In the patient with germline *WWOX* P252A and P282A variants, WES revealed 11.5 somatic non‐synonymous single‐base substitutions (SBS)/Mb in the thyroid mixed tumor. Using a 96‐substitution classification, the somatic mutational signature was displayed, and the predominant mutations were C>T transitions (Figure S6A, Supporting Information). The SBS mutational signature significantly resembled SBS signatures 1, 6, 10b, and 87, which were reported by COSMIC.^[^ ^37^ ^]^ The mutational signature of the thyroid mixed tumor was mostly associated with SBS signature 1, which is related to the age at diagnosis. SBS10b is related to polymerase epsilon exonuclease domain mutations. The other associated signatures, 6 and 87, are characterized by defective DNA mismatch repair (Figure S6B, Supporting Information). Besides, WES analysis revealed the presence of 164 insertion‐deletions (InDels) per megabase and identified 5+ base insertions as the predominant InDel mutations in the thyroid mixed tumor (Figure S6C, Supporting Information). The InDel mutational signature exhibited a strong resemblance to COSMIC signature ID8, which displays a correlation with the age of cancer diagnosis. Additional comparable signatures included COSMIC signatures ID2, ID4, ID5, ID10, ID12, ID14, and ID17. Among these InDel signatures, the presence of signature ID2 has been linked to defective DNA mismatch repair (Figure S6D, Supporting Information). These data suggest that the thyroid mixed tumor with *WWOX* P252A and P282A variants is associated with the deficiency of DNA damage repair.

**Figure 5:**
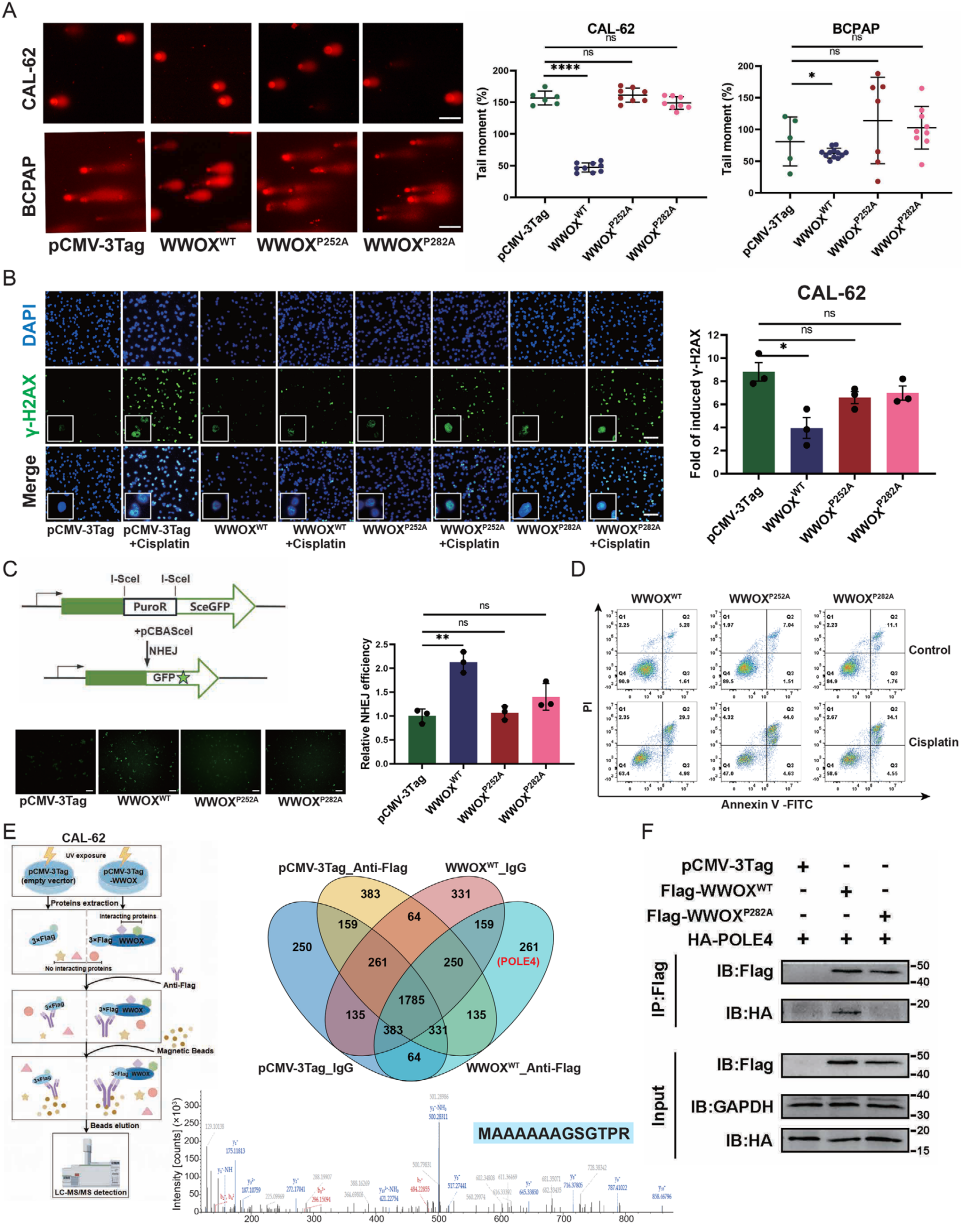
The ability of DNA damage repair of WWOX^P252A^ and WWOX^P282A^ mutants is impaired. A) Representative fluorescence images of the comet assay from UV‐irradiated CAL‐62 and BCPAP cells. Decreased tail moment indicates fewer DNA strand breaks in cells with stable over‐expression of wild‐type WWOX, but not in cells with stable over‐expression of WWOX^P252A^ or WWOX^P282A^ mutants. Tail moment was quantified. Data are shown as mean ± SEM from three independent experiments (*n* = 3, Student's t‐test). Scale bar: 40 µm. B) Representative images of γ‐H2AX (green) in CAL‐62 cells treated with and without 20 µM cisplatin for 24 h. Nucleus is stained with DAPI (blue). The induction of γ‐H2AX foci by cisplatin is significantly reduced in CAL‐62 cells with stable over‐expression of wild‐type WWOX, but not in CAL‐62 cells with stable over‐expression of WWOX^P252A^ or WWOX^P282A^ mutants. An inset in each panel shows an enlarged view of the γ‐H2AX foci. The number of γ‐H2AX‐positive cells was counted by ImageJ and divided by the total number of nuclei counted in the same view. The fold change in γ‐H2AX‐positive cells induced by cisplatin was calculated relative to the corresponding control group. Data are shown as mean ± SEM from three independent experiments (*n* = 3, Student's t‐test). Scale bar: 50 µm. C) NHEJ reporter system shows that the repair efficiency of NHEJ is increased by the over‐expression of wild‐type WWOX, but not by the over‐expression of WWOX^P252A^ or WWOX^P282A^ mutant. The schematic diagram of the NHEJ reporter system shows that functional GFP expression is formed only upon precise NHEJ‐mediated repair. CAL‐62 cells with stable over‐expression of wild‐type WWOX, WWOX^P252A,^ or WWOX^P282A^ mutant were transiently transfected with NHEJ reporter and I‐SceI endonuclease‐expressing plasmids for 48 h. GFP fluorescence was imaged, and GFP‐positive cells were quantified to show NHEJ efficiency. Data are shown as mean ± SEM from three biological replicates (*n* = 3, Student's t‐test). Scale bar: 50 µm. D) Flow cytometry shows that cisplatin treatment (20 µM, 24 h) induces fewer apoptotic cells in CAL‐62 cells with stable over‐expression of wild‐type WWOX, compared to CAL‐62 cells with stable over‐expression of WWOX^P252A^ or WWOX^P282A^ mutant. E) IP‐MS analysis identifies POLE4 interacting with WWOX. Schematic workflow (drawn by Figdraw) of IP‐MS shows that wild‐type WWOX is immunoprecipitated from CAL‐62 cells with stable over‐expression of WWOX using anti‐FLAG antibody, and then co‐precipitated proteins were analyzed by mass spectrometry. A Venn diagram shows proteins co‐precipitated by anti‐FLAG antibody or IgG control in CAL‐62 cells with stable transfection of pCMV‐3Tag empty vector or Flag‐tagged WWOX^WT^ vector. POLE4 is only co‐precipitated by anti‐FLAG antibody in CAL‐62 cells stably expressing WWOX^WT^ protein. LC/MS spectrum of a peptide (sequence: MAAAAAAGSGTPR) from POLE4 is shown. F) Co‐IP analysis shows the interaction of WWOX and POLE4. HEK293T cells were co‐transfected with Flag‐tagged wild‐type (WWOX^WT^) or mutant (WWOX^P282A^) vector and HA‐tagged POLE4 vector. After UV exposure, cell lysates were immunoprecipitated with anti‐FLAG antibody, followed by a western blot for POLE4 (HA‐tagged).

To verify the effect of WWOX^P252A^ and WWOX^P282A^ mutants on DNA single‐strand damage repair, we used the comet assay to detect DNA damage induced by UV exposure. Stable expression of wild‐type WWOX significantly attenuated UV‐induced tail moment in CAL‐62 and BCPAP cells; in contrast, cells expressing the WWOX^P252A^ or WWOX^P282A^ mutant exhibited DNA damage levels similar to the empty vector control (**Figure** **5**A). Furthermore, to detect whether mutated WWOX reduces the ability of DNA double‐strand damage repair, we used immunofluorescence staining to measure the number of phosphorylated histone H2AX (γH2AX) positive foci induced by chemo‐drug cisplain. Consistent with DNA single‐strand damage repair, cisplatin‐induced γH2AX foci were significantly attenuated in CAL‐62 cells stably expressing wild‐type WWOX. WWOX^P252A^ and WWOX^P282A^ mutants did not decrease the γH2AX foci (Figure 5B). To investigate whether WWOX participates in DNA damage repair directly, an NHEJ‐mediated EGFP reporter was used to measure the repair rates of I‐SceI‐induced DSBs. NHEJ‐mediated EGFP reporter was transfected into CAL‐62 cells stably expressing with empty vector, wild‐type WWOX, WWOX^P252A,^ or WWOX^P282A^ mutant. As shown in Figure 5C, the over‐expression of wild‐type WWOX increased the repair efficiency of NHEJ, while the repair efficiency of NHEJ in CAL‐62 cells stably expressed with WWOX^P252A^ or WWOX^P282A^ mutant was the same as that in CAL‐62 cells stably expressed with an empty vector. Previous studies have shown that cancer cells with a deficiency of DNA damage repair were sensitive to chemotherapy.^[^ ^38^ ^]^ Indeed, we found that chemo‐drug cisplatin induced more Annexin V‐positive apoptotic cells in CAL‐62 cells stably expressed with WWOX^P252A^ and WWOX^P282A^ mutants, compared with CAL‐62 cells stably expressed with wild‐type WWOX (Figure 5D; Figure S7, Supporting Information). These data demonstrate that WWOX participates in DNA damage repair directly; however, WWOX^P252A^ and WWOX^P282A^ mutants lose the ability to repair DNA damage.

Previous studies have indicated that WWOX was involved in DNA damage repair by interacting with other proteins, such as BRCA1, ATM, and ATR, etc.^[^ ^39^ ^]^ To identify new WWOX interaction partners associated with DNA damage repair, CAL‐62 cells with the stable overexpression of wild‐type WWOX were treated with UV, and protein lysates were subjected to immunoprecipitation with FLAG antibody, and then the proteins that interacted with FLAG‐WWOX were identified by liquid chromatography‐tandem mass spectrometry (LC‐MS/MS) (Figure 5E). LC‐MS/MS identified a total of 261 proteins specifically bound to the wild‐type WWOX (Table S6, Supporting Information). We subsequently performed Gene Ontology (GO) and KEGG pathway enrichment analyses on the 261 putative WWOX interactors. As shown in Figure S8 and Table S7 (Supporting Information), the enrichment of terms related to RNA processing and metabolism, which is consistent with a previous study,^[^ ^40^ ^]^ was observed. Notably, the thyroid hormone signaling pathway was also significantly enriched. Among these WWOX interacting partners, POLE4 was the only candidate protein involved in DNA repair (Figure 5E). POLE4, a subunit of the DNA polymerase epsilon complex, is involved in UV‐induced nucleotide excision repair.^[^ ^41^ ^]^ We co‐transfected POLE4 and WWOX‐expressing vectors in HEK293T cells. After UV treatment, the co‐IP further indicated that there was an interaction between wild‐type WWOX and POLE4. However, the WWOX^P282A^ mutant appeared to lose its interaction with POLE4 (Figure 5F). The interaction between POLE4 and the WWOX^P252A^ mutant was also markedly reduced, likely due to the low abundance of the unstable WWOX^P252A^ mutant (Figure S9, Supporting Information).

Furthermore, we modeled the 3D structures of the wild‐type WWOX protein and its P252A and P282A mutants using PyMOL. The geometric and topological properties of the wild‐type and mutated structures were analyzed using CASTp 3.0 software. Quantitative structural analysis indicated an enlargement of the substrate‐binding pockets in both WWOX^P252A^ and WWOX^P282A^ mutants, which was characterized by greater surface area and volume compared to the wild‐type (Figure S10A, Supporting Information). Conformational changes in both WWOX^P252A^ and WWOX^P282A^ mutants include shortened hydrogen bonds, resulting in reduced protein rigidity and increased conformational flexibility (Figure S10B, Supporting Information). This structural plasticity likely underlies the observed pocket enlargement. We next simulated the binding of WWOX (including wild type, P252A, and P282A mutants) and the partners (HSC70 and POLE4) by molecular docking. The docking results showed that compared with wild‐type WWOX, the P252A mutation enhanced the binding energy of WWOX to HSC70 (Figure S11A, Supporting Information). The P282A mutation in the WWOX protein reduced the binding energy for POLE4, compared to the wild‐type WWOX (Figure S11B, Supporting Information). Therefore, the molecular docking results suggest that the P252A mutation enhances WWOX binding to HSC70, whereas the P282A mutation reduces its affinity for POLE4.

In summary, WWOX^P252A^ and WWOX^P282A^ mutants lose the ability to repair DNA damage. POLE4 is a newly identified interaction partner with WWOX, and their interaction may be involved in UV‐induced nucleotide excision repair.

### 2.5 Low Expression of WWOX Protein is Associated with EMT and Aggressive Phenotype in Thyroid Cancer

**Figure 6:**
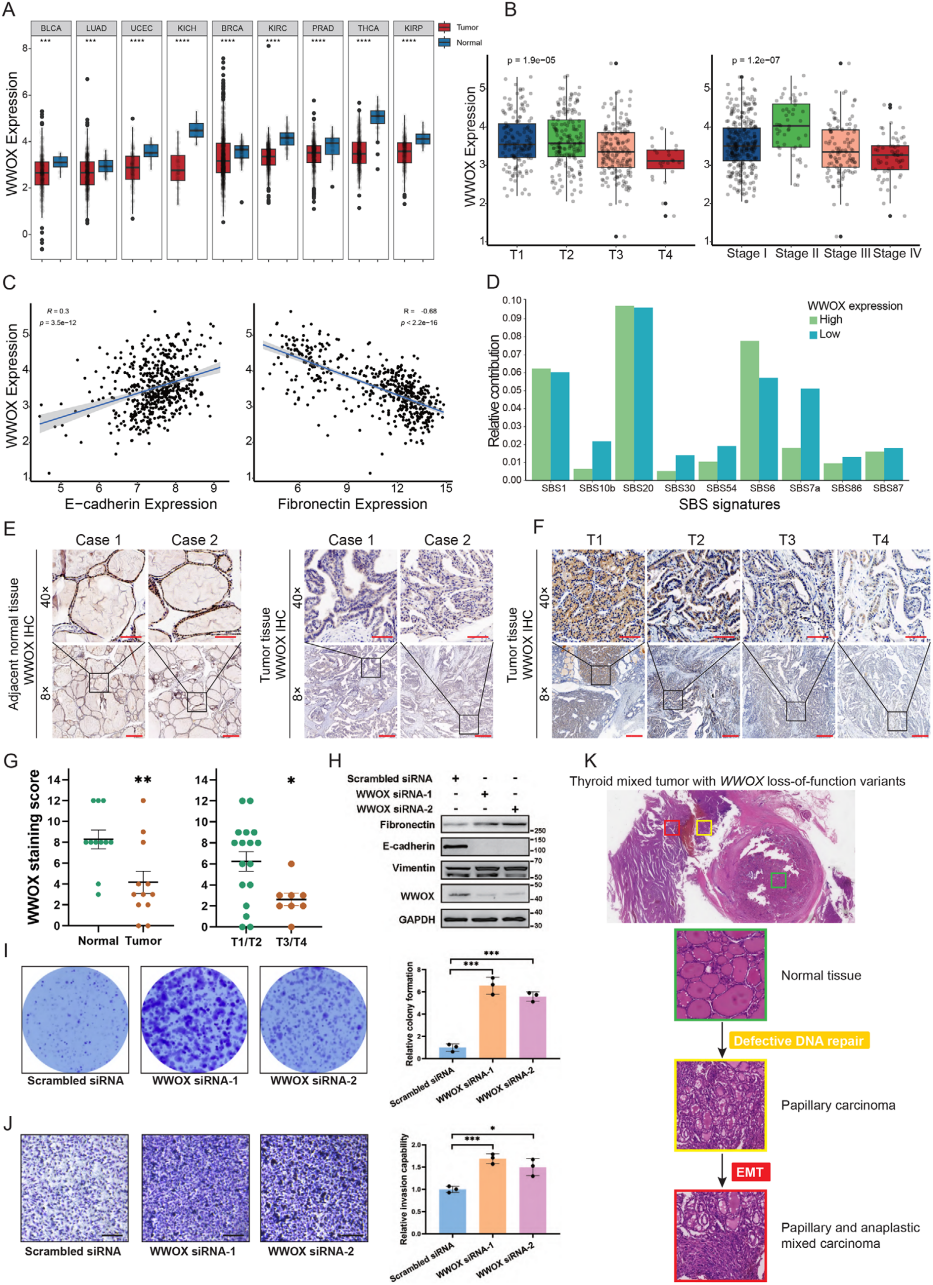
Low expression of WWOX protein is associated with EMT and aggressive phenotype in thyroid cancer. A) TCGA data show that WWOX mRNA levels are significantly down‐regulated in tumor tissues compared to normal tissues in 9 cancer types (Wilcoxon test). BLCA: Bladder urothelial carcinoma, LUAD: Lung adenocarcinoma, UCEC: Uterine corpus endometrial carcinoma, KICH: Kidney chromophobe, BRCA: Breast invasive carcinoma, KIRC: Kidney renal clear cell carcinoma, PRAD: Prostate adenocarcinoma, THCA: Thyroid carcinoma, KIRP: Kidney renal papillary cell carcinoma. B) TCGA data show that WWOX mRNA levels were significantly lower in advanced thyroid tumors with T3/T4 grades or III/IV stages (Kruskal‐Wallis test). C) TCGA data shows that WWOX mRNA expression has a significant positive correlation with E‐cadherin mRNA expression, and a negative correlation with Fibronectin mRNA expression (Pearson's correlation). D) Histogram shows the contributions of COSMIC mutational signatures in thyroid tumors with high and low WWOX expression from the TCGA dataset. Signatures SBS10b (Polymerase epsilon exonuclease domain mutations), SBS7a (UV exposure), and SBS30 (defective DNA base excision repair) are enriched in thyroid tumors with low WWOX expression. E) Representative IHC staining of WWOX in tumor and adjacent normal tissues from FFPE specimens of thyroid cancer patients. Scale bar: 60 µm for 8×, 400 µm for 40×. F) Representative IHC staining of WWOX in tumor tissues with different T grades (T1‐T4). Scale bar: 60 µm for 8×, 400 µm for 40×. G) IHC scores of WWOX in tumor and adjacent normal tissues from 12 thyroid cancer patients (left panel, Mann‐Whitney U test). IHC scores of WWOX in tumor tissues with T1/T2 and T3/T4 grades from 25 thyroid cancer patients (right panel, Mann‐Whitney U test). H) Western blot indicates that knockdown of WWOX using two independent siRNAs for 48 h in CAL‐62 cells results in down‐regulation of E‐cadherin, and up‐regulation of Vimentin and Fibronectin. I) Colony formation assay indicates that the ability of colony formation is significantly increased in WWOX siRNA‐transfected CAL‐62 cells compared to scrambled siRNA‐transfected CAL‐62 cells. The number of colonies was quantified. Data are shown as mean ± SEM from three independent experiments (*n* = 3, Student's t‐test). J) Transwell invasion assay indicates that the invasion is significantly increased in WWOX siRNA‐transfected CAL‐62 cells compared to scrambled siRNA‐transfected CAL‐62 cells. The number of invaded cells was quantified. Data are shown as mean ± SEM from three independent experiments (*n* = 3, Student's t‐test). K) Schematic illustration of thyroid mixed tumor formation. The H&E staining of the thyroid mixed tumor includes normal thyroid tissue (green panel), papillary carcinoma (yellow panel), and anaplastic carcinoma (red panel). Defective DNA repair promotes the initiation of papillary thyroid carcinoma. Furthermore, EMT promotes the dedifferentiation of papillary thyroid carcinoma into aggressive anaplastic thyroid carcinoma.

To investigate whether the impairment of WWOX promotes cancer progression, we first analyzed the expression of WWOX in 60 cancer types using the dataset of The Cancer Genome Atlas (TCGA). The result showed that the expression of WWOX mRNA was significantly lower in 9 cancer types, including thyroid, bladder, lung, endometrium, kidney, breast, and prostate cancers (**Figure** **6**A). The expression of WWOX was significantly lower in thyroid cancer with late stages (Figure 6B). In thyroid cancer, WWOX expression correlated positively with the expression of epithelial marker E‐cadherin, while WWOX expression correlated negatively with the expression of mesenchymal marker fibronectin (Figure 6C). Based on the mean value of WWOX expression, thyroid tumors from the TCGA database were classified into two groups: one group with high WWOX expression (*n* = 203) and the other group with low WWOX expression (*n* = 294). Signatures SBS10b (Polymerase epsilon exonuclease domain mutations), SBS7a (UV exposure), and SBS30 (defective DNA base excision repair) are higher in thyroid tumors with low WWOX expression (Figure 6D). We also detected the expression of the WWOX protein using the collected clinical specimens of thyroid cancer. IHC staining showed that the expression of WWOX protein was significantly lower in tumor tissues, compared with adjacent normal tissues (Figure 6E,G). The expression of WWOX protein was significantly lower in advanced thyroid tumors (T3/T4) (Figure 6F,G).

Western blot indicated that WWOX knockdown by siRNA induced epithelial‐mesenchymal transition, as shown by the increased expression of mesenchymal markers (Fibronectin and Vimentin) and the decreased expression of epithelial marker (E‐cadherin) (Figure 6H). We further validated that down‐regulation of WWOX expression enhanced the abilities of colony formation and invasion in CAL‐62 cells (Figure 6I,J).

In summary, these data indicate that the reduced WWOX protein expression promotes EMT and aggressive phenotype in thyroid cancer. Furthermore, our findings suggest that thyroid mixed tumor may originate from a defective DNA repair mechanism, leading to the initiation of papillary thyroid carcinoma; while EMT could promote the dedifferentiation of papillary thyroid carcinoma into aggressive anaplastic thyroid carcinoma (Figure 6K).

## 3 Discussion

Germline loss‐of‐function mutations in the *WWOX* gene have been reported to be associated with a severe early‐onset epileptic encephalopathy called WOREE syndrome.^[^ ^27^ ^]^ Case‐control association studies have reported that some germline *WWOX* variants were related to cancer susceptibility.^[^ ^42^ ^]^ However, it is poorly understood whether and how these *WWOX* variants lose the function of a tumor suppressor. In this study, we identified two *WWOX* germline variants (P252A and P282A) in a young male patient with a rare thyroid mixed tumor. By in vitro and in vivo study, we demonstrated that WWOX^P252A^ and WWOX^P282A^ mutant proteins lost the activity of tumor suppression and DNA damage repair. We examined the mechanisms and found that the two variants impaired WWOX function through accelerated protein degradation and disrupted protein‐protein interaction. To our knowledge, this study provides the first functional evidence of *WWOX* variants, elucidating whether and how *WWOX* variants lose cancer‐protective function.

Although WWOX was initially discovered as a putative tumor suppressor, no case report has documented the development of cancer in patients with WOREE syndrome. This may be due to a high frequency of premature death by the age of 1–4 years in the severe neurological disorder of WOREE syndrome.^[^ ^28^ ^]^ In this study, the cancer patient harboring germline homozygous *WWOX* P252A and P282A variants did not suffer from WWOX‐related nervous system disease. We speculate that the complete functional loss of the WWOX mutated protein is expected to lead to severe WOREE syndrome‐related phenotypes. However, WWOX^P252A^ and WWOX^P282A^ mutants may have some residual protein function to maintain the development of the neurological system.

This is the first study to indicate that the *WWOX* P252A variant is associated with cancer. We further validated that the WWOX^P252A^ mutant lost the function of a tumor suppressor by in vitro and in vivo studies. Our study indicated that the WWOX^P252A^ mutant protein was more susceptible to degradation compared with the wild‐type WWOX protein. WWOX^P252A^ mutant protein underwent rapid turnover, thereby exhibiting low expression. The impaired expression of WWOX^P252A^ mutant directly correlated with a marked attenuation of its tumor‐suppressive capacity. This loss‐of‐function mechanism due to mutant protein instability via rapid degradation was also found in other tumor suppressors. Previous studies have shown that the *MEN1* E235K variant was established as an oncogenic driver in gastroenteropancreatic neuroendocrine tumors, where mutant menin underwent enhanced proteasomal‐independent degradation, ablating its tumor‐suppressive activity.^[^ ^43^ ^]^ In a family with Li‐Fraumeni Syndrome, Lee et al. found a germline missense R145W mutation in the *CHK2* gene. CHK2^R145W^ mutant encoded an unstable protein, which was degraded rapidly by the proteosome pathway.^[^ ^44^ ^]^ Functional characterization of rare germline missense *BRCA1* variants also identified a subset (p.M297V, p.D1152N, p.L52F, p.L1439F, and p.G890R) that exhibited reduced protein stability.^[^ ^45^ ^]^ Niemann‐Pick type C disease, a fatal neurodegenerative disorder, was caused by loss‐of‐function mutations in the *NPC1* gene. The most common disease‐causing *NPC1* I1061T variant produced a misfolded protein in the endoplasmic reticulum (ER), which was rapidly degraded by the proteasome and ER autophagy.^[^ ^46^ ^]^ In our study, degradation of the WWOX^P252A^ mutant protein occurred via chaperone‐mediated autophagy in the lysosome, not via the proteasome pathway. Previous studies have indicated that mutant p53 was degraded through chaperone‐mediated autophagy in a lysosome‐dependent fashion.^[^ ^47^ ^]^ The WWOX protein contains a recognition sequence (KFERQ‐like motif) for HSC70, a chaperone protein involved in chaperone‐mediated autophagy.^[^ ^48^ ^]^ The KFERQ‐like motif (LRSVQ, amino acids 187‐191) in WWOX is situated in close proximity to the site of the P252A variant. Structural analysis reveals that the P252A variant reduces protein rigidity and increases conformational flexibility, suggesting that this enhanced structural plasticity underlies the observed pocket enlargement. Therefore, conformational change in WWOX^P252A^ mutant likely exposes the KFERQ‐like motif for HSC70 binding.

Previous studies have indicated that K63‐linked polyubiquitylation is linked to chaperone‐mediated autophagy.^[^ ^48^ ^]^ K63 polyubiquitination can enhance the binding affinity of substrate proteins to HSC70. For instance, a previous study has demonstrated that HIF1α is targeted for degradation by chaperone‐mediated autophagy through a mechanism dependent on K63‐linked ubiquitination. The K63‐linked ubiquitination of HIF1α by the E3 ligase STUB1 facilitates its degradation by chaperone‐mediated autophagy, as the ubiquitin chain enhances the recognition of HSC70.^[^ ^49^ ^]^ Indeed, we found that the WWOX^P252A^ mutant protein undergoes K63‐linked polyubiquitination. This modification may enhance its interaction with HSC70, leading to its subsequent transport to the lysosome for degradation.

*WWOX* P282A variant, a SNP rs3764340, has been reported to be associated with the susceptibility to many malignant tumors, including gastric cancer, lung cancer, oral cancer, osteosarcoma, thyroid carcinoma, hepatocellular carcinoma, and esophageal cancer.^[^ ^50^ ^]^ Proline‐282‐alanine substitution may cause a change in WWOX protein structure, thereby affecting the function of the tumor suppressor.^[^ ^30^ ^]^ Our study provides the first experimental evidence, demonstrating that the WWOX^P282A^ mutant protein lost the ability of a tumor suppressor. Nevertheless, the precise molecular mechanism underlying the loss‐of‐function of the *WWOX* P282A variant is still unknown. By immunoprecipitation coupled with mass spectrometry, we found a novel WWOX interaction partner, POLE4, but the WWOX^P282A^ mutant protein was not able to bind to POLE4. POLE4 and POLE3 are accessory subunits of DNA polymerase epsilon (POLϵ), which mediates efficient and accurate DNA replication and repair. Dysfunction of the POLE complex is associated with genetic instability and cancer.^[^ ^51^ ^]^ Bellelli et al. have shown that *Pole4* knockout in mice induced inefficient origin activation, replicative damage, and genome instability.^[^ ^52^ ^]^ UV induces bulky DNA adducts, which are repaired by nucleotide excision repair. DNA polymerase epsilon is involved in the last step of nucleotide excision repair for new DNA strand synthesis. Therefore, WWOX may recruit POLE4 through the interaction to participate in nucleotide excision repair induced by UV. But the WWOX^P282A^ mutant protein fails to bind to POLE4, preventing the accurate repair of DNA breaks and ultimately leading to genomic instability and increased cancer risk. This is consistent with the mutational signature analysis of the thyroid tumor. In the thyroid mixed tumor of the patient, the somatic mutation signature significantly resembled SBS10b, which is related to polymerase epsilon exonuclease domain mutations. Using the TCGA dataset, SBS10b and UV‐related SBS7a signatures were enriched in thyroid tumors with low WWOX expression. However, the functional relevance of WWOX‐POLE4 interaction in nucleotide excision repair remains to be elucidated, necessitating further investigation to determine whether this protein complex directly modulates DNA damage recognition or repair fidelity in genomic maintenance.

Accumulating evidence has demonstrated that WWOX plays a key role in DNA damage response. Abu‐Odeh et al. have shown that WWOX, via its WW1 domain, interacts with ATM, which was involved in DNA DSB repair.^[^ ^17^ ^]^ In response to DNA damage, phosphorylation of ATM promoted ITCH‐mediated ubiquitination and nucleus translocation of WWOX, which then interacted with ATM to enhance its activation. WWOX‐loss cells had defects in ATM and γ‐H2AX recruitment to DNA damage sites.^[^ ^17^ ^]^ WWOX was also involved in DNA SSB repair through ATR activation.^[^ ^18^ ^]^ Schrock et al. have indicated that mouse embryo fibroblast cells from *Wwox*‐knockout mice exhibited mild chromosome instability in genomes. WWOX deficiency disrupted DNA DSB repair, enhancing reliance on error‐prone repair.^[^ ^19^ ^]^ Bidany‐Mizrahi et al. have found that mice with conditional knockout of both *Wwox* and *Brca1* genes developed basal‐like mammary tumors, exhibiting impaired DSB repair.^[^ ^39^ ^]^ These previous studies indicated that WWOX deficiency impaired the repair of DNA DSB and SSB, thereby providing a selective merit to acquire additional cancer‐associated driver mutations in neoplastic transformation. Our study indicates that WWOX^P252A^ and WWOX^P282A^ mutant proteins lose the ability of DNA DSB and SSB repair, suggesting a driver role for carcinogenesis through genomic instability.

WWOX is highly expressed in several hormone‐related organs, including the prostate, mammary gland, and ovary. The high expression of WWOX in these tissues suggests its potential involvement in regulating hormone metabolism.^[^ ^2^ ^]^ A previous study has shown that *Wwox* knockout mice exhibit impaired steroidogenesis in the reproductive system.^[^ ^53^ ^]^ As shown in Figure 6A, analysis of TCGA data shows that WWOX is highly expressed in the thyroid gland (a major hormone‐secreting organ). Our IP‐MS analysis identified putative WWOX interactors enriched in the thyroid hormone signaling pathway. These data further imply an important role of WWOX in thyroid hormone signaling.

In this study, we identify two germline homozygous *WWOX* variants (P252A and P282A) in a young male patient with a rare thyroid mixed tumor. We provide the first functional evidence that both variants completely abrogate WWOX's tumor‐suppressive activity. Mechanistically, WWOX^P252A^ mutant undergoes accelerated degradation via chaperone‐mediated autophagy in the lysosome, directly linking protein instability to loss‐of‐function. Furthermore, both WWOX mutants exhibit defective DNA damage repair capacity, thereby probably predisposing carriers to cancer through genomic instability. Finally, WWOX deficiency promotes EMT, which induces the malignant transformation.

## 4 Experimental Section

### Human Specimens

Formalin‐Fixed and Paraffin‐Embedded (FFPE) specimens of the thyroid mixed tumor and adjacent normal tissue were collected from a Chinese male patient who underwent surgery at the First Affiliated Hospital of USTC. Peripheral blood leukocytes of the patient's son and wife were also collected to extract genomic DNA. The study was approved by the Clinical Research Ethics Committee of the First Affiliated Hospital of USTC (Approval number: 2024‐RE‐296). For IHC analysis of WWOX expression, FFPE specimens of paired tumor and adjacent normal tissues were collected from 25 thyroid cancer patients who underwent surgery at Hefei Cancer Hospital of the Chinese Academy of Sciences. The study was approved by the Ethics Committee of Hefei Institutes of Physical Science (Approval number: SWYX‐Y‐2021‐12). All tumor and non‐tumor tissue samples were confirmed by two experienced pathologists. Written informed consent to participate in the study was provided.

### Genomic DNA Extraction

For FFPE samples, genomic DNA of the patient's tumor and adjacent normal tissues was extracted by the GeneRead DNA FFPE Kit (#180134, QIAGEN, Hilden, Germany) according to the manufacturer's instructions. For blood samples, genomic DNA was extracted using the TIANamp Genomic DNA Kit (#DP304, Tiangen, Beijing, China) according to the manufacturer's instructions. DNA concentration was measured using a Qubit Fluorometer (Thermo Fisher Scientific, Waltham, MA, USA).

### WES and Variants Calling

Libraries of WES were prepared using KAPA Hyper Prep Kit (#KK8504, KAPA Biosystems, Wilmington, MA, USA), and exome capture was carried out using SeqCap EZ Human Exome Library v3.0 (Roche, CA, USA). WES was performed on the Illumina NovaSeq platform (Illumina, CA, USA). The quality of sequencing reads was checked using FastQC (version 0.11.7), and then adaptors and low‐quality bases were trimmed using Trim Galore (version 0.6.3). Reads were mapped to the hg19 human reference genome using the Burrows–Wheeler Alignment tool (BWA, version 0.7.15). Samtools (version 1.7.0) was used to convert SAM alignment files to BAM format. Variant calling of germline single‐nucleotide variants (SNVs) and insertions‐deletions (InDels) was performed using HaplotypeCaller of GATK (version 4.1.8). Mutect2 (v2.2) was used to call somatic mutations and InDels. Called variants were annotated with ANNOVAR software.

### Cell Lines

Human thyroid cancer cell lines CAL‐62 (#CC2306) and BCPAP (#CC2304), and HEK293T cell line (#CC4003) were purchased from the Cellcook Biotech (Guangzhou, China). CAL‐62 and HEK293T cells were cultured in DMEM medium (#10‐013‐CVRC, Corning, NY, USA) supplemented with 10% fetal bovine serum (#FSP500, ExCell, Shanghai, China) and 1% penicillin‐streptomycin (#SV30010, Hyclone, Logan, UT, USA). BCPAP cells were cultured in RPMI 1640 complete medium (#10‐013‐CVR, Corning, NY, USA). All the cells were maintained at 37 °C under a humidified incubator with 5% CO_2_.

### Plasmids, siRNA, Antibodies, and Drugs

Wild‐type *WWOX* full‐length cDNA (NM_016373) was cloned into pCMV‐3Tag expression vector (#240195, Agilent, Santa Clara, CA, USA). *WWOX* P252A, P282A single and double mutations in the pCMV‐Flag‐WWOX vector were generated using a mutagenesis kit (#E0554, NEB, Ipswich, England) based on the manufacturer's instructions. *WWOX* P252A, P282A single and double mutations in the vector were confirmed by Sanger sequencing. NHEJ reporter plasmids, EJ2GFP‐puro (#44025) and I‐SceI endonuclease expression vector pCBASceI (#26477) were from Addgene (Watertown, MA, USA). The expression vectors of wild‐type Ubiquitin pRK5‐HA‐Ubiquitin (#P1750) and mutant Ubiquitin pRK5‐HA‐Ubiquitin‐K48R (#P51109), pRK5‐HA‐Ubiquitin‐K63R (#P51110) were purchased from MiaoLingBio, Wuhan, China. POLE4‐expressing vector pcDNA‐HA‐POLE4 was purchased from Youbio biotechnology (Changsha, China).

WWOX siRNA oligos were purchased from GenePharma (Shanghai, China). The sense and antisense sequences are as below:

WWOX siRNA #1

Sense: 5′‐GAAGCAUUCAAGGCCAAGATT‐3′

Antisense: 5′‐UCUUGGCCUUGAAUGCUUCTT‐3′

WWOX siRNA #2

Sense: 5′‐CCUGGAAAUAUGAUGUACUTT‐3′

Antisense: 5′‐AGUACAUCAUAUUUCCAGGTT‐3′.

Anti‐FLAG antibody (clone M2, #F1804) was purchased from Sigma–Aldrich (St. Louis, MO, USA). Anti‐WWOX antibody (#ab238144) was purchased from Abcam (Cambridge, UK). Anti‐PARP (#9542), GAPDH (#2118), HA (#3724), E‐Cadherin (#3195), Vimentin (#5741), Fibronectin (#26836), Ki‐67 (#9449), and γH2AX (#9718) antibodies were purchased from Cell Signaling Technology (Danvers, MA, USA). HSC70 antibody (#AF1132) was purchased from Beyotime (Shanghai, China). Secondary antibodies for immunofluorescence staining were obtained from Invitrogen (Carlsbad, CA, USA): donkey anti‐rabbit IgG conjugated to Alexa Fluor 488 (#A21206) and donkey anti‐mouse IgG conjugated to Alexa Fluor 568 (#A11004). Secondary antibodies for western blot were obtained from Cell Signaling Technology (Danvers, MA, USA): HRP‐linked anti‐mouse IgG (#7076S) and HRP‐linked anti‐rabbit IgG (#7074S).

Cisplatin (#HY‐17394), MG132 (#HY‐13259), Chloroquine (#HY‐17589A), NH_4_Cl (#HY‐Y1269), and 3‐MA (#HY‐Y1269) were purchased from MedChemExpress (MCE, Shanghai, China).

### Cell Transfection

Cells were transfected with siRNA using Lipofectamine 2000 (#11668019, Invitrogen, Carlsbad, CA, USA), according to the manufacturer's instructions. Cells were transfected with the plasmids using Effectene Transfection Reagent (#301425, QIAGEN, Hilden, Germany).

### The Generation of Cell Lines with Stable Expression of Wild‐Type and Mutant WWOX

CAL‐62 and BCPAP cells were transfected with pCMV‐Flag empty vector, pCMV‐Flag‐WWOX^WT^, pCMV‐Flag‐WWOX^P252A,^ and pCMV‐Flag‐WWOX^P282A^ vectors. After 48 h of transfection, the culture medium was replaced with a fresh medium containing G418 (#345810, Sigma, St. Louis, MO, USA) at 800 µg mL^−1^ concentration for selection. Individual G418‐resistant colonies were picked using pipette tips for growing and validating the expression of Flag‐tagged WWOX by western blot.

### Western Blot and Immunoprecipitation

For western blot analysis, cells were lysed in lysis buffer (50 mm Tris‐HCl pH 7.5, 1% NP‐40, 0.1% SDS, 0.5% sodium deoxycholate, 0.15 m NaCl, 50 mm NaF, 1 mm EDTA, 1 mm Na_3_VO_4_, 1 mm DTT) with protease inhibitor (#EA0006, SparkJade, Qingdao, China) for 30 min on ice. Protein lysis was collected by centrifugation at 13300 × rpm for 10 min at 4 °C, and quantified by a BCA assay (#P0009, Beyotime, Shanghai, China). 20 µg of protein samples were separated on SDS‐PAGE, and then transferred to a PVDF membrane (#IPVH00010, Merck Millipore, Billerica, MA, USA). After blocking with 5% non‐fat milk for 2 h, the membrane was blotted with the primary antibodies overnight at 4 °C, and then followed by incubation with horseradish peroxidase‐conjugated secondary antibodies at room temperature for 1.5 h. Blots were developed by using the chemiluminescence system (NcmECL Ultra, Nanjing, China).

For immunoprecipitation (IP), cells were lysed using IP lysis buffer (#P0013, Beyotime, Shanghai, China). Whole‐cell protein extracts were incubated with the corresponding antibody overnight at 4 °C. After the addition of protein A/G magnetic beads (#P2108, Beyotime, Shanghai, China), the incubation was continued for an additional 2 h at room temperature. Then, the beads were resuspended in 2×sample buffer and boiled for 10 min prior to western blot analysis.

### Immunohistochemistry (IHC) Staining

IHC staining was carried out according to a previous study.^[^ ^54^ ^]^ Briefly, tumor tissues were dehydrated through a graded ethanol series (70–100%), cleared in xylene, and infiltrated with molten paraffin wax before being embedded into paraffin blocks using standard protocols. Sections of paraffin‐embedded tissues were incubated at 63 °C for 3 h. Subsequently, they were dewaxed in xylene and rehydrated through a descending ethanol series (100%, 95%, 85%, and 75%; 5 min each). Antigen retrieval was performed using citric acid buffer. After blocking with peroxidase blocking solution for 30 min, the slides were incubated overnight at 4 °C in a humidified chamber with the following primary antibodies diluted at specified ratios: anti‐WWOX (1:200), anti‐Ki67 (1:500), anti‐Vimentin (1:200), and anti‐E‐cadherin (1:200). The sections were then incubated for 45 min with a secondary antibody (KIT‐5020, MaxVision‐HRP mouse/rabbit, Maxim, Fuzhou, China). Staining was developed using a DAB kit (ZLI‐9018, Zhongshan Golden Bridge, Beijing, China), followed by counterstaining with hematoxylin, dehydration, and mounting. The results were evaluated by two experienced pathologists, based on staining intensity (0, 1+, 2+ and 3+) and the percentage of positive cells, categorized as follows: 0 (0%), 1 (1–25%), 2 (26–50%), 3 (51–75%) and 4 (76–100%).

### Cell Proliferation and Colony Formation Assay

Cell proliferation was assessed using cell counting and CellTiter‐Glo Luminescent Assay. For cell counting, cells were seeded in 6‐well plates (5000 cells per well). After incubation, the cell suspension was stained with 0.4% (w/v) trypan blue solution (#T8154, Sigma–Aldrich, St. Louis, MO, USA). The stained cells were immediately loaded onto a hemocytometer. Viable (unstained) cells were counted under an inverted phase‐contrast microscope (Leica Microsystems, Wetzlar, Germany). For the CellTiter‐Glo Luminescent Assay, cells were seeded in a 96‐well plate (1 × 10^3^ cells per well). After the incubation, 20 µL of Cell Titer‐Glo reagent (#G7572, Promega, Madison, WI, USA) was added to the wells. The plate was incubated for 6–8 min, and the luminescent signal was recorded using a CMAX microplate reader (Molecular Devices, San Jose, CA, USA).

To examine the ability of colony formation, cells were seeded in 6‐well plates (1000 cells per well) and allowed to grow for an additional 18 days. Transiently transfected cells were seeded at a density of 3000 cells per well in 6‐well plates and cultured for 12 days post‐transfection. Colonies were fixed with paraformaldehyde (4%) for 20 min and stained with crystal violet (1%) for 15 min. Colony numbers were quantified using ImageJ software (NIH, Bethesda, MD, USA).

### Cell Invasion and Migration Assay

To detect the ability of cell invasion, the Matrigel invasion assay was conducted. Cells (1–2 × 10⁵ cells) in serum‐free medium were seeded into the upper chamber of a transwell insert (#3422, Corning, NY, USA) pre‐coated with Matrigel (#356231, BD Biosciences, Sparks, NJ, USA). The lower chamber was filled with 600 µL complete medium. After incubation for 24 h, the invaded cells in the lower surface of the insert were fixed with 4% paraformaldehyde and then stained with 1% crystal violet. The invaded cells were imaged using a microscope (Leica Microsystems, Wetzlar, Germany).

To detect the ability of cell migration, a wound healing assay was conducted. Cells (5 × 10⁵ cells per well) were seeded in 6‐well plates, scratched with a pipette tip, and gently washed to remove the detached cells. After incubation in serum‐free medium, migration in the scratched area was recorded at 0 and 20 h. Migration distance was measured at five fixed points, and wound closure was quantified by comparing the initial and final images.

### Immunofluorescence Staining

Cells were seeded onto the Millicell EZ SLIDE 12‐well glass slides (#PEZGS0496, Millipore, MA, USA). After treatment, cells were washed with cold PBS and fixed in 4% paraformaldehyde for 20 min at 37 °C. Fixed cells were washed with PBS three times and permeabilized with 0.5% Triton X‐100 for 10 min. Subsequently, cells were blocked in blocking buffer (2% bovine serum albumin and 5% fetal bovine serum in TBST) for 1 h and incubated with the primary antibody. Following an overnight incubation at 4 °C, slides were washed with TBST and incubated with fluorescence‐conjugated secondary antibody at room temperature in the dark for 1 h. After washing, the mounting medium with DAPI (#ab104139, Sigma, St. Louis, MO, USA) was applied onto the slides. The slides were imaged under a ZEISS fluorescence microscope (Oberkochen, Germany) and quantified using ImageJ software (NIH, Bethesda, MD, USA).

### Cycloheximide (CHX) Chase Assay

To determine the half‐life of wild‐type WWOX and the two mutated WWOX proteins, a CHX chase assay was performed. Briefly, cells were treated with 50 µg mL^−1^ CHX (#HY‐12320, MCE, Shanghai, China) to inhibit protein synthesis, and cell protein lysate was collected at 0, 2, 4, 6, 8, and 10 h post‐treatment, and then subjected to western blot analysis. Band intensities were quantified using ImageJ software (NIH, Bethesda, MD, USA).

### Reverse Transcription Real‐Time quantitative Polymerase‐Chain Reaction (RT‐qPCR)

Total RNA was extracted by using TRIzol Reagent (#DP451, Tiangen, Beijing, China) according to the manufacturer's instructions. Total RNA was subjected to reverse transcription using the TransScript First‐Strand cDNA Synthesis SuperMix kit (#AT301‐03, TransGen, Beijing, China). Universal SYBR Green Master Mix Kit (#Q341‐02, Vazyme, Nanjing, China) was applied for qPCR assay. The relative mRNA levels were normalized to the β‐actin level, and 2^−ΔΔCt^ values were calculated.

The primers for the detection of WWOX mRNA expression were as follows:

Sense: 5′‐CATCTGGGGCACTTCTACCTTGT‐3′

Antisense: 5′‐GAGAGGCGACTGAAGTCCAGT‐3′

The primers used for β‐actin mRNA expression were as follows:

Sense: 5′‐CATGTACGTTGCTATCCAGGC‐3′

Antisense: 5′‐CTCCTTAATGTCACGCACGATGC‐3′

### Comet Assay

Cells were treated with UV light for 30 min, and then cultured for 6 h. The treated cells were harvested and mixed with 0.5% low‐melting‐point soft agar gel. Alkaline lysis buffer (2.5 m NaCl, 100 mm Na_2_EDTA, 10 mm Tris, 1% TritonX‐100, pH 10) was used to release DNA at 4 °C for 2 h. DNA was separated using alkaline electrophoresis buffer (10 m NaOH and 200 mm Na_2_EDTA) at 25 V for 30 min. Cells were neutralized with Tris‐HCl (pH 7.5) and then stained using 0.1% Super Red (#BS354B, Biosharp, Beijing, China). Finally, the cells were imaged under a ZEISS fluorescence microscope (Oberkochen, Germany). The quantification of tail DNA was performed using ImageJ software (NIH, Bethesda, MD, USA).

### NHEJ Reporter Assays

CAL‐62 cells with stable expression of wild‐type and mutant WWOX were seeded in 6‐well plates (2×10⁵ cells per well). Cells were co‐transfected with 1 µg I‐SceI (pCBAScel) and NHEJ reporter plasmid (EJ2GFP‐puro) with Effectene Transfection Reagent. 48 h after transfection, cells were imaged under a ZEISS fluorescence microscope (Oberkochen, Germany). NHEJ efficiency was calculated as the percentage of GFP+ cells.

### Immunoprecipitation Coupled with LC‐MS/MS Analysis

CAL‐62 cells stably overexpressed with pCMV‐Flag‐WWOX (wild type) or pCMV‐Flag (empty vector) were treated with UV for 30 min, and then cultured for 12 h. Cells were lysed with IP lysis buffer (#P0013, Beyotime, Beijing, China) supplemented with protease inhibitors (#P0015, Beyotime, Beijing, China). Cell lysates were incubated with anti‐Flag (#F1804, Sigma, St. Louis, MO, USA) or IgG (#A7028, Beyotime, Shanghai, China) in a rotating incubator overnight at 4 °C. Cell lysates were then incubated with protein A+G magnetic beads (#P2108, Beyotime, Shanghai, China) for 1 h at room temperature and washed three times with 1×TBS buffer. The beads with immunoprecipitated proteins were sent to the MS facility (iProteome, Shanghai, China). The liquid chromatography‐tandem mass spectrometry (LC‐MS/MS) analysis and data processing were done by the MS specialist from the MS facility (iProteome, Shanghai, China).

### Cell Apoptosis

After treatment, cells were harvested and stained with 5 µL of Annexin V‐FITC for 15 min and 5 µL of PI for 5 min using an Apoptosis Detection Kit (BD Pharmingen, San Diego, CA, USA) at room temperature in the dark. After adding 400 µL of 1×binding buffer, the stained cells were subjected to flow cytometry analysis (CytExpert, Beckman Coulter, Brea, CA, USA). The percentage of apoptotic cells was calculated using FlowJo software (BD Pharmingen, Ashland, OR, USA).

### Mouse Experiments

4–5 weeks old female Athymic Nude mice were purchased from Vital River (Beijing, China). Mice were raised under 12‐h light/dark cycle and with a standard diet at the specific pathogen‐free‐graded animal house at Hefei Cancer Hospital of the Chinese Academy of Sciences. Cancer cells (2×10^6^ cells per 100 µl medium) were mixed with 100 µl Matrigel (#47743‐720, BD Biosciences, Franklin, NJ, USA) and then subcutaneously injected into each flank of the mouse. Tumor volume was measured at four‐day intervals using a caliper and calculated based on the equation:

(1) V=L×W×W/2

where L is the longest diameter, and W is the shortest diameter.

At the end of the experiment, the mice were euthanized by CO_2_ asphyxiation. The tumors were dissected and weighed, and then fixed in 4% paraformaldehyde for 24 h. H&E and IHC staining were performed as previously described.^[^ ^54^ ^]^ All mouse experiments were performed according to a protocol (Approval number: DWLLPF‐2023112301) approved by the Institutional Animal Care and Use Committee of Hefei Cancer Hospital of Chinese Academy of Sciences.

### Statistical Analysis

The data were analyzed using GraphPad Prism version 8.0 (GraphPad Software, Inc., La Jolla, CA, USA). All quantitative data are shown as mean ± SEM from three biological replicates. Comparisons between two groups were analyzed using a two‐tailed unpaired Student's t test or Wilcoxon test, depending on the normality of data distribution. Comparisons of ordered categorical data between two groups were performed using the non‐parametric Mann–Whitney U test. Comparisons among multiple groups (different stages of thyroid cancer) were analyzed using the Kruskal–Wallis test. Correlation analysis was performed using Pearson's correlation method. *p* < 0.05 was considered as statistical significance (*p* < 0.05^*^, *p* < 0.01^**^, *p* < 0.001^***^, *p* < 0.0001^****^). The COSMIC mutational signatures analysis was performed using the R (version 4.1.3) packages “MutationalPatterns” and “FitMS”.

### Ethics Approval

The FFPE specimens of the thyroid tumor and adjacent normal tissue were collected from a Chinese male patient who underwent surgery at the First Affiliated Hospital of USTC. Peripheral blood leukocytes of the patient's son and wife were also collected. The study was approved by the Clinical Research Ethics Committee of the First Affiliated Hospital of USTC (Approval number: 2024‐RE‐296). FFPE specimens of paired tumor and adjacent normal tissues were collected from 25 thyroid cancer patients who underwent surgery at Hefei Cancer Hospital of the Chinese Academy of Sciences. The study was approved by the Ethics Committee of Hefei Institutes of Physical Science (Approval number: SWYX‐Y‐2021‐12). Written informed consent to participate in the study was provided. All mouse experiments were performed according to a protocol (Approval number: DWLLPF‐2023112301) approved by the Institutional Animal Care and Use Committee of Hefei Cancer Hospital of Chinese Academy of Sciences.

## Conflict of Interest

The authors declare no conflict of interest.

## Author Contributions

B.H., H.W., and X.Z. designed the study. X.Z., Z.W., and Y.W. performed the experiments. J.Q. and J.W. performed the bioinformatic analysis. X.Z., A.X., and Z.H. collected the clinical samples. X.Z. and B.H. wrote the manuscript. X.Z. and B.H. edited the manuscript. All authors reviewed the manuscript.

## Supporting information



Supporting Information

Supplemental Table 6

Supplemental Table 7

## Data Availability

The WES sequencing data of the patient are not publicly available due to privacy concerns, ethical considerations, and legal requirements. All other data supporting the findings of this study are available in the Supporting Information.
