# Supplementary material for: Genetic and Functional Evidence Links Germline Biallelic Inactivating Variants in WWOX to Histological Mixed‐Type Thyroid Cancer
Source: Adv Sci (Weinh). 2025 Oct 22;13(1):e07602. doi: 10.1002/advs.202507602 (PMC12767083; doi:10.1002/advs.202507602)
Supplement: Supplementary file 1 — Supporting Information [file ADVS-13-e07602-s001.docx]

**Supplementary material**

**Genetic and functional evidence links germline biallelic inactivating variants in *WWOX* to histological mixed-type thyroid cancer**

Xiaopeng Zhang^1, 2^, Jian Qi^1, 2^ Jialiang Wang^1^, Zhipeng Wang^1, 2^, Yongguang Wang^1^, Zongtao Hu^1^, Ao Xu^3, #^, Bo Hong^1, 2, #^, Hongzhi Wang^1, 2,^ ^#^

^1^ Hefei Cancer Hospital of CAS, Institute of Health and Medical Technology, Hefei Institutes of Physical Science, Chinese Academy of Sciences (CAS), Hefei 230031, Anhui, China

^2^ Science Island Branch, Graduate School of University of Science and Technology of China, Hefei 230026, Anhui, China

^3^ Department of Pathology, The First Affiliated Hospital of USTC, Division of Life Sciences and Medicine, University of Science and Technology of China (USTC), Hefei 230036, Anhui, China

^#^**Denotes co-corresponding authorship**

Corresponding to:

**Bo Hong**: Hefei Cancer Hospital of CAS, Institute of Health and Medical Technology, Hefei Institutes of Physical Science, Chinese Academy of Sciences (CAS), Hefei 230031, Anhui, China; E-mail: bhong@hmfl.ac.cn

**Hongzhi Wang**: Hefei Cancer Hospital of CAS, Institute of Health and Medical Technology, Hefei Institutes of Physical Science, Chinese Academy of Sciences (CAS), Hefei 230031, Anhui, China; E-mail: wanghz@hfcas.ac.cn

**Ao Xu**: Department of Pathology, The First Affiliated Hospital of USTC, Division of Life Sciences and Medicine, University of Science and Technology of China (USTC), Hefei 230036, Anhui, China; E-mail: aoxuhf@ustc.edu.cn

**Supplementary Figure 1:**


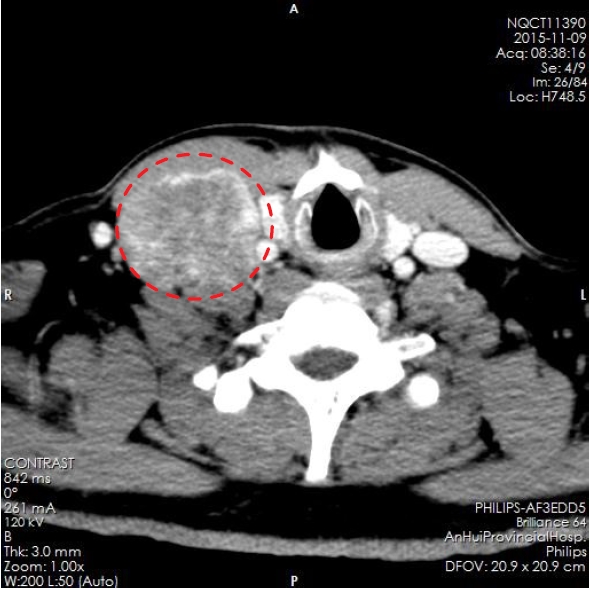


**Supplementary Figure 1.** **A contrast-enhanced CT scan of the patient shows a thyroid tumor located in the right neck region, demarcated by a red dashed circle.**

**Supplementary Table 1: Statistics overview of whole exome sequencing**

| **Samples** | **Total reads** | **Raw size (Gb)** | **Mean depth (X)** | **Coverage rate (%)** | **Coverage (>=10x)** | **Coverage (>=30x)** | **Coverage (>=100x)** |
| --- | --- | --- | --- | --- | --- | --- | --- |
| **Normal** | **124078476** | **11.2** | **35** | **99.80%** | **97.95%** | **88.02%** | **39.26%** |
| **Tumor** | **307527565** | **30.6** | **135** | **99.97%** | **99.64%** | **98.05%** | **85.43%** |

**Supplementary Table 2: The predicted damaging germline variants to impact protein function**

| **Gene** | **mRNA accession number** | **Nucleotide change** | **Protein change** | **SIFT prediction** | **Polyphen prediction** | **MutationTaster prediction** | **MutationAssessor prediction** | **LRT prediction** | **FATHMM prediction** | **gnomAD**  **allele frequency** | **Genotype** |
| --- | --- | --- | --- | --- | --- | --- | --- | --- | --- | --- | --- |
| ***AK2*** | **NM_001319142** | **c.476A>T** | **Y159F** | Deleterious | Probably damaging | Disease causing | Medium impact | Deleterious | Deleterious | **0.0009** | Hetero |
| ***HGD*** | **NM_000187** | **c.1027A>C** | **M343L** | Deleterious | Possibly damaging | Disease causing | Medium impact | Deleterious | Deleterious | **0.0002** | Hetero |
| ***ZGRF1*** | **NM_001350397** | **c.5236C>T** | **P1746S** | Deleterious | Possibly damaging | Disease causing | Low impact | Deleterious | Deleterious | **0.0025** | Hetero |
| ***KIF13B*** | **NM_015254** | **c.3275G>A** | **R1092H** | Deleterious | Probably damaging | Disease causing | Medium impact | Deleterious | Deleterious | **0.00009** | Hetero |
| ***AQP7*** | **NM_001318156** | **c.411C>A** | **N137K** | Deleterious | Probably damaging | Disease causing | Low impact | Deleterious | Deleterious | **0.0022** | Hetero |
| ***WWOX*** | **NM_016373** | **c.754C>G** | **P252A** | Deleterious | Possibly damaging | Disease causing | Low impact | Deleterious | Deleterious | **0.0063** | Homo |
| ***PTPRT*** | **NM_007050** | **c.4221C>G** | **F1407L** | Deleterious | Probably damaging | Disease causing | Medium impact | Deleterious | Deleterious | **0.0002** | Hetero |

Hetero: Heterozygous variant, Homo: Homozygous variant

**Supplementary Table 3: The frameshift and nonsense germline variants**

| **Gene** | **mRNA accession number** | **Nucleotide change** | **Protein change** | **Mutation type** | **Genotype** |
| --- | --- | --- | --- | --- | --- |
| *IGSF3* | NM_001007237 | c.G1724A | p.W575* | Nonsense | Hetero |
| *TCHHL1* | NM_001008536 | c.C1966T | p.Q656* | Nonsense | Hetero |
| *ANKRD36C* | NM_001310154 | c.1930_1931insAT | p.S644Yfs* | Frameshift | Hetero |
| *ANKRD36C* | NM_001310154 | c.1925_1926del | p.Q642Pfs* | Frameshift | Hetero |
| *ANKRD36C* | NM_001310154 | c.G1177T | p.E393* | Nonsense | Hetero |
| *ANKRD36* | NM_001354587 | c.3460_3461del | p.S1154Wfs* | Frameshift | Hetero |
| *XIRP2* | NM_001199144 | c.C3643T | p.R1215* | Nonsense | Hetero |
| *ZNF717* | NM_001128223 | c.T1284A | p.C428* | Nonsense | Hetero |
| *POLN* | NM_181808 | c.2509delC | p.Q837Sfs* | Frameshift | Hetero |
| *STATH* | NM_001009181 | c.C129A | p.Y43* | Nonsense | Hetero |
| *SLC9B1* | NM_001100874 | c.C913T | p.R305* | Nonsense | Hetero |
| *SDHA* | NM_001330758 | c.1701_1702del | p.L568Efs* | Frameshift | Hetero |
| *SLC25A2* | NM_031947 | c.G778T | p.E260* | Nonsense | Hetero |
| *FAM8A1* | NM_016255 | c.C1015T | p.R339* | Nonsense | Hetero |
| *HLA-DRB5* | NM_002125 | c.44_45insCT | p.T16* | Nonsense | Hetero |
| *MYO6* | NM_001300899 | c.2743dupA | p.Q918Tfs* | Frameshift | Hetero |
| *FOXO3* | NM_001455 | c.1141dupG | p.L382Afs* | Frameshift | Hetero |
| *HGC6.3* | NM_001129895 | c.501_502del | p.L168Afs* | Frameshift | Hetero |
| *SPDYE3* | NM_001004351 | c.C1627T | p.R543* | Nonsense | Hetero |
| *MUC3A* | NM_005960 | c.1437_1438del | p.T480Lfs* | Frameshift | Hetero |
| *MUC3A* | NM_005960 | c.1440_1441insTG | p.S481Cfs* | Frameshift | Hetero |
| *PCMTD1* | NM_001286782 | c.784dupA | p.I262Nfs* | Frameshift | Hetero |
| *PCMTD1* | NM_001286782 | c.C526T | p.R176* | Nonsense | Hetero |
| *ANKRD20A3* | NM_001012419 | c.C791G | p.S264* | Nonsense | Hetero |
| *SPATA31C1* | NM_001145124 | c.224_225insGC | p.C76Rfs* | Frameshift | Hetero |
| *SPATA31C1* | NM_001145124 | c.226_227insCATCTTGTCTCCCAGC | p.C76Sfs* | Frameshift | Hetero |
| *OR13C8* | NM_001004483 | c.C26A | p.S9* | Nonsense | Hetero |
| *CYP2C18* | NM_000772 | c.T204A | p.Y68* | Nonsense | Hetero |
| *CYP2C19* | NM_000769 | c.G636A | p.W212* | Nonsense | Hetero |
| *MUC5B* | NM_002458 | c.3292_3293insGGCCGGGGGGCGTTTCTCTGGG | p.S1098Wfs* | Frameshift | Hetero |
| *GXYLT1* | NM_001099650 | c.T699A | p.Y233* | Nonsense | Hetero |
| *TMBIM4* | NM_001282609 | c.485dupT | p.L162Ffs* | Frameshift | Hetero |
| *LRRIQ1* | NM_001079910 | c.5096dupA | p.N1702Kfs* | Frameshift | Hetero |
| *CAMKK2* | NM_001270486 | c.1614_1615insAAAA | p.G539Kfs* | Frameshift | Hetero |
| *SKA3* | NM_001166017 | c.208delC | p.Q70Kfs* | Frameshift | Hetero |
| *PABPC3* | NM_030979 | c.819_822del | p.T274Nfs* | Frameshift | Hetero |
| *CCDC168* | NM_001146197 | c.16123dupA | p.I5375Nfs* | Frameshift | Hetero |
| *LRRC74A* | NM_194287 | c.485delA | p.Y162Sfs* | Frameshift | Hetero |
| *OR4M2* | NM_001004719 | c.C382T | p.R128* | Nonsense | Hetero |
| *MNS1* | NM_018365 | c.605dupA | p.Q203Afs* | Frameshift | Hetero |
| *ACSM2A* | NM_001308169 | c.C106T | p.R36* | Nonsense | Hetero |
| *POLR2C* | NM_032940 | c.C337T | p.R113* | Nonsense | Hetero |
| *KRT13* | NM_002274 | c.1247dupA | p.R417Afs*82 | Frameshift | Hetero |
| *CDC27* | NM_001293091 | c.C115T | p.Q39* | Nonsense | Hetero |
| *TAC4* | NM_001077503 | c.196_199del | p.G66Hfs* | Frameshift | Hetero |
| *MUC16* | NM_024690 | c.40675_40676del | p.S13559Pfs* | Frameshift | Hetero |
| *MUC16* | NM_024690 | c.40668delT | p.S13559Afs* | Frameshift | Hetero |
| *MUC16* | NM_024690 | c.38938_38939insAA | p.P12980Qfs* | Frameshift | Hetero |
| *MUC16* | NM_024690 | c.38935_38936del | p.A12979Pfs* | Frameshift | Hetero |
| *OR7G3* | NM_001001958 | c.C658T | p.R220* | Nonsense | Hetero |
| *ZNF28* | NM_001369762 | c.469_470del | p.M157Efs* | Frameshift | Hetero |
| *SIRPB1* | NM_001083910 | c.392_393del | p.V131Gfs* | Frameshift | Hetero |
| *SIRPB1* | NM_001083910 | c.388delG | p.D130Tfs* | Frameshift | Hetero |
| *ARSD* | NM_001669 | c.G992A | p.W331* | Nonsense | Hetero |
| *FAM104B* | NM_001166699 | c.C238T | p.Q80* | Nonsense | Hetero |

Hetero: Heterozygous variant

**Supplementary Table 4: *WWOX* germline variants detected in the patient**

| **Gene** | **mRNA accession number** | **Nucleotide change** | **Protein change** | **SIFT prediction** | **Polyphen prediction** | **MutationTaster prediction** | **MutationAssessor prediction** | **LRT prediction** | **FATHMM prediction** | **gnomAD**  **allele frequency** | **Genotype** |
| --- | --- | --- | --- | --- | --- | --- | --- | --- | --- | --- | --- |
| ***WWOX*** | **NM_016373** | **c.754C>G** | **P252A** | Deleterious | Possibly damaging | Disease causing | Low impact | Deleterious | Deleterious | **0.0063** | Homo |
| ***WWOX*** | **NM_016373** | **c.844C>G** | **P282A** | Tolerated | Probably damaging | Polymorphism automatic | Low impact | Deleterious | Deleterious | **0.0733** | Homo |

Homo: Homozygous variant

**Supplementary Table 5: Somatic driver mutations detected in the thyroid mixed tumor**

| **Gene** | **mRNA accession number** | **Nucleotide change** | **Protein change** | **Allele frequency (%)** | **Gene function** |
| --- | --- | --- | --- | --- | --- |
| ***MSH6*** | **NM_000179** | **c.3254delC** | **T1085fs** | **7.6** | **DNA repair** |
| ***ARID1B*** | **NM_017519** | **c.5365C>T** | **R1789*** | **7.8** | **Epigenetic regulation** |
| ***ATP2A1*** | **NM_001286075** | **c.2082delC** | **R694fs** | **15.8** | **ATPase** |
| ***TP53*** | **NM_001126115** | **c.451C>T** | **R151C** | **6.3** | **Cell apoptosis** |
| ***BTK*** | **NM_001287345** | **c.1169C>T** | **P390L** | **10.3** | **Signaling transduction** |

**Supplementary Figure 2:**


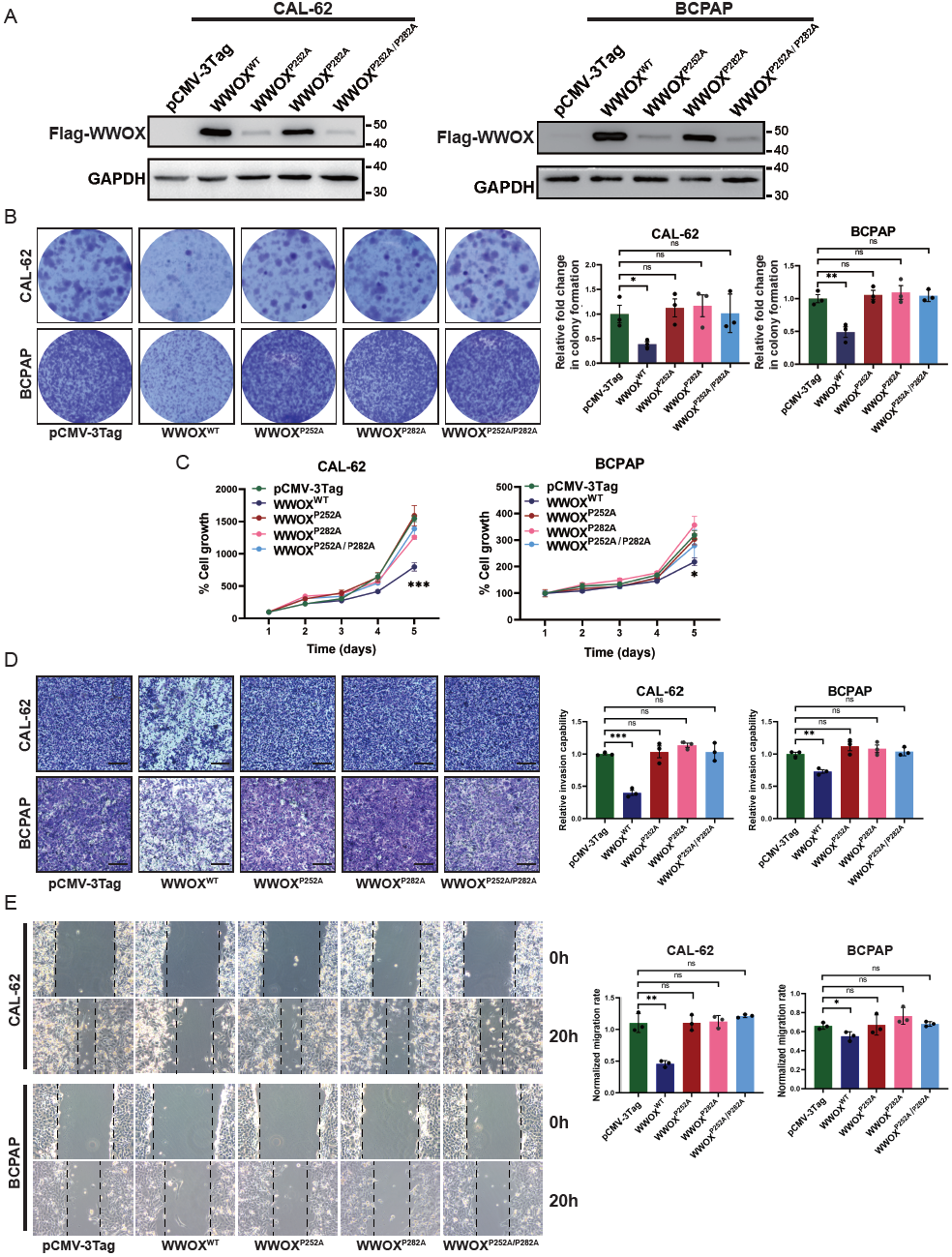


**Supplementary Figure 2. The WWOX double mutant (WWOX^P252A/P282A^) loses the tumor-suppressive function.** (**A**) Western blot validates the stable over-expression of Flag-tagged wild-type (WT), P252A or P282A single mutant, and P252A/P282A double mutant WWOX proteins in CAL-62 and BCPAP thyroid cancer cells. (**B**) Colony formation assay indicates that the ability of colony formation is significantly reduced in CAL-62 and BCPAP cells stably over-expressing wild-type WWOX protein, but not in cells stably over-expressing WWOX^P252A^ or WWOX^P282A^ single mutant, and WWOX^P252A/P282A^ double mutant. The colonies were quantified relative to empty vector and shown by mean ± SEM from three independent experiments (n=3, Student’s t-test). (**C**) The CellTiter-Glo Luminescent assay shows that stable over-expression of wild-type WWOX significantly inhibits the proliferation of CAL-62 and BCPAP cells, whereas over-expression of WWOX^P252A^, WWOX^P282A^, and WWOX^P252A/P282A^ mutants do not exert such an inhibitory effect. Error bars represent mean ± SEM (n=3, Student’s t-test). (**D**) Matri-gel invasion assay indicates that stable over-expression of wild-type WWOX significantly inhibits the invasion of CAL-62 and BCPAP cells, but WWOX^P252A^, WWOX^P282A^ and WWOX^P252A/P282A^ mutants lose the inhibitory ability of cell invasion. The invaded cells were quantified and shown by mean ± SEM from three independent experiments (n=3, Student’s t-test). (**E**) Wound-healing assay indicates that stable over-expression of wild-type WWOX significantly inhibits the migration of CAL-62 and BCPAP cells, whereas WWOX^P252A^, WWOX^P282A^ and WWOX^P252A/P282A^ mutants lose the inhibitory ability of cell migration. Wound closure at 20 hours (20h) was normalized to the initial wound area at 0 hour (0h), and quantified as a migration rate. The data is shown by mean ± SEM from three independent experiments (n=3, Student’s t-test).

**Supplementary Figure 3:**


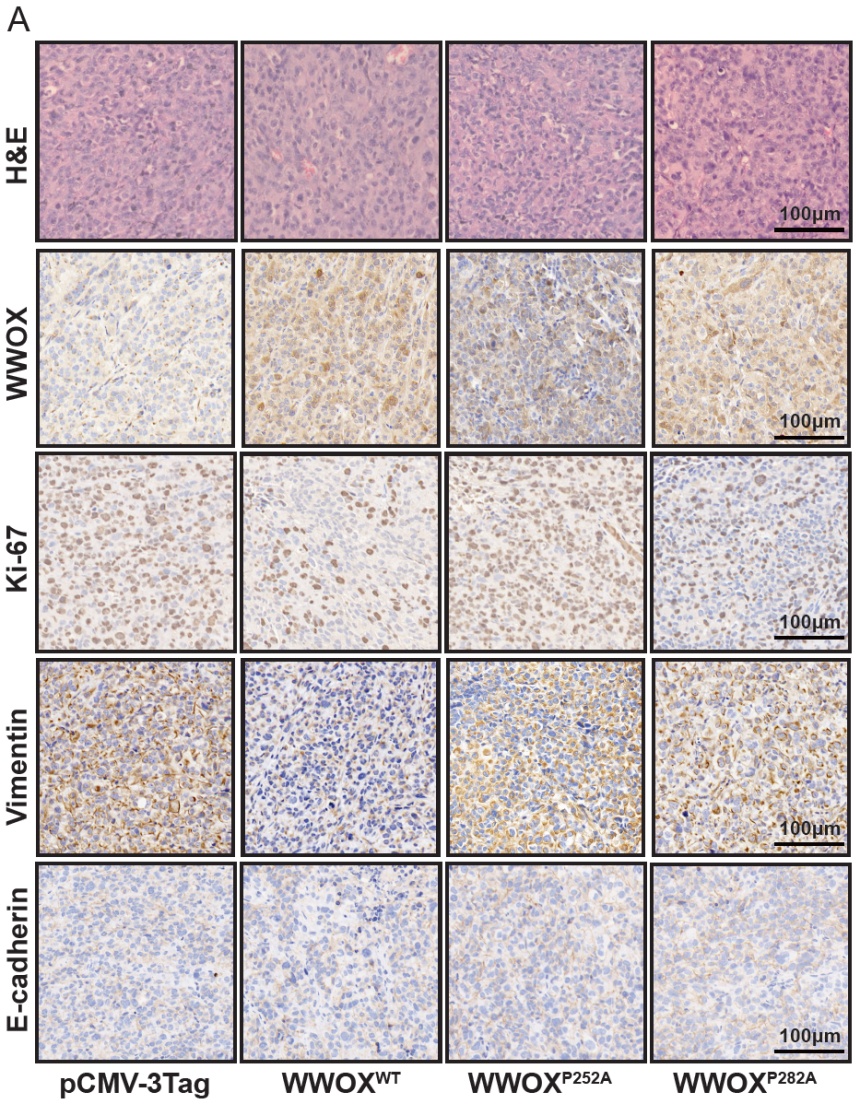


**Supplementary Figure 3. H&E and IHC staining images (40× magnification) of tumor tissues obtained from xenografts in different mouse groups.**

**Supplementary Figure 4:**


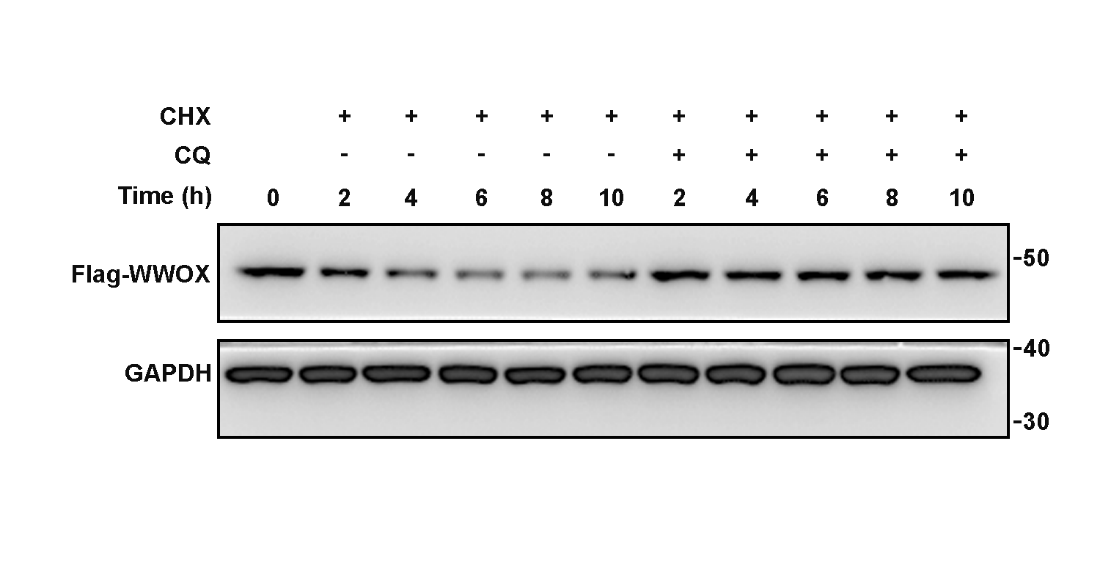


**Supplementary Figure 4.** **Chloroquine (CQ) prevents the degradation of WWOX^P252A^ mutant protein in the presence of CHX.** CAL-62 cells stably expressing the WWOX^P252A^ mutant protein were treated with CHX (50 µg/mL), either alone or in combination with CQ (25 µM), for the indicated durations. The expression of Flag-WWOX was detected by western blot.

**Supplementary Figure 5:**


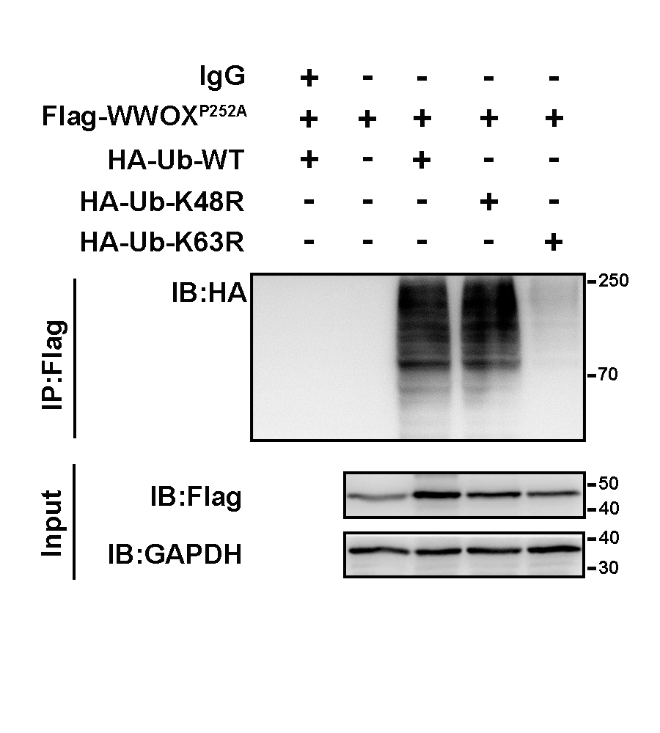


**Supplementary Figure 5. Co-IP indicates that WWOX^P252A^ mutant protein is conjugated with K63-linked polyubiquitylation chain.** HEK293T cells were co-transfected with a Flag-tagged mutant WWOX plasmid (Flag-WWOX^P252A^) along with plasmids encoding HA-tagged wild-type ubiquitin (HA-Ub-WT), the K48R ubiquitin mutant (HA-Ub-K48R), or the K63R ubiquitin mutant (HA-Ub-K63R). Immunoprecipitation (IP) was performed using anti-FLAG or control IgG antibody, followed by immunoblotting (IB) with an anti-HA antibody to detect ubiquitin. The K63R ubiquitin mutant reduces WWOX polyubiquitination of WWOX^P252A^ protein, compared to wild-type ubiquitin or the K48R mutant.

**Supplementary Figure 6:**


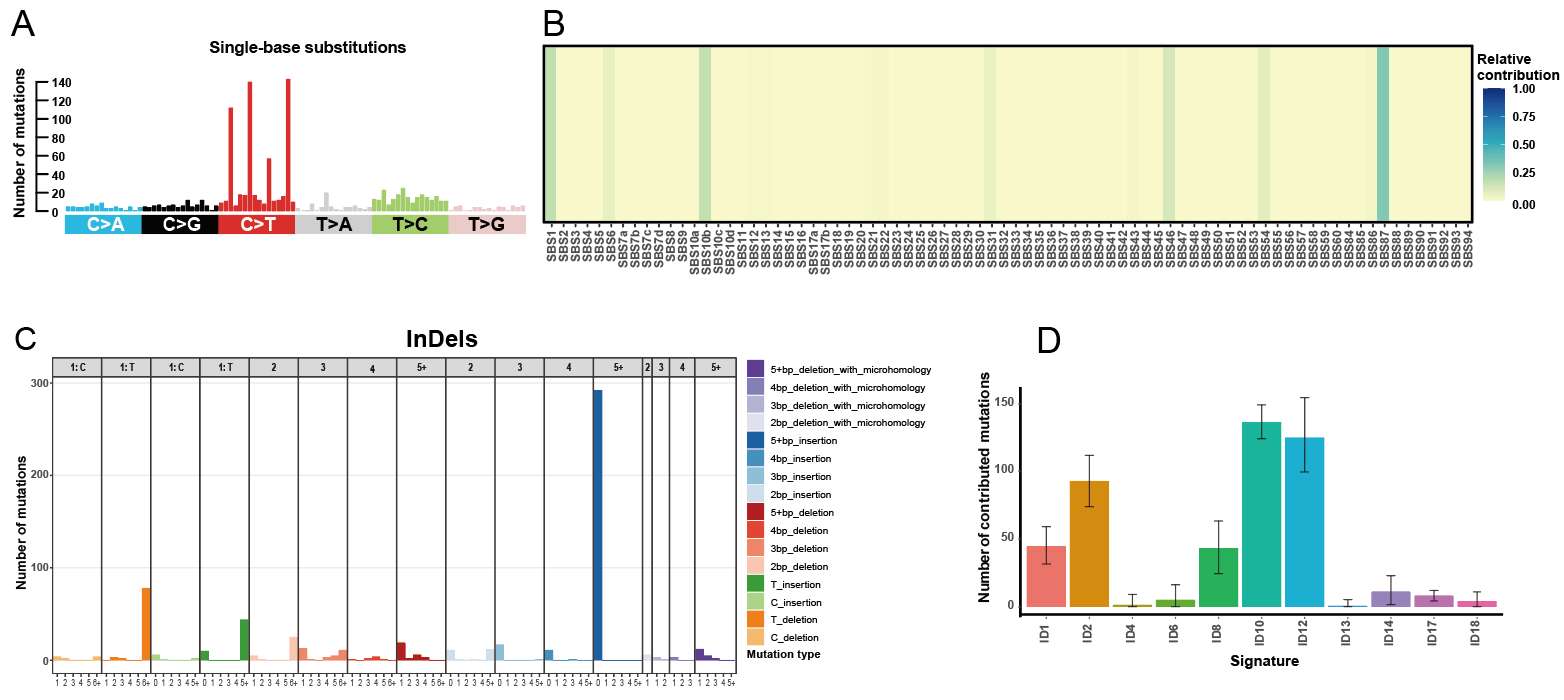


**Supplementary Figure 6. The somatic SBS and InDel mutational signatures of the thyroid mixed tumor. (A)** The somatic SBS mutational signature of the thyroid mixed tumor. The SBS signature is identified using the 96 substitution classification. **(B)** The contribution of the SBS mutational signature. **(C)** The somatic InDel mutational signature of the thyroid mixed tumor. The InDel signature is identified using 83 different types considering size, nucleotides affected and presence on repetitive and/or microhomology regions. (**D**) The contribution of the InDel mutational signature.

**Supplementary Figure 7:**


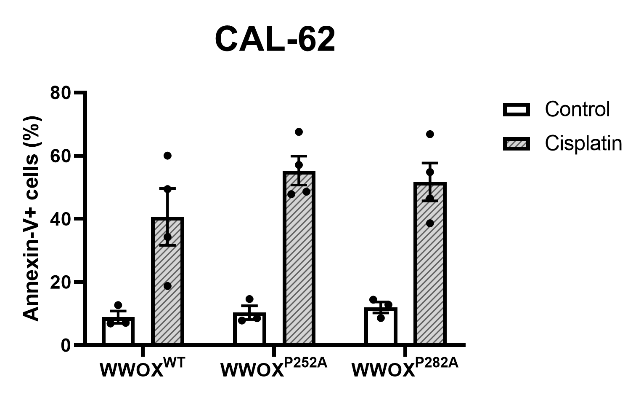


**Supplementary Figure 7. Quantitative analysis of apoptotic cells based on the percentage of Annexin V-positive cells by flow cytometry.** CAL-62 cells stably expressing wild-type, P252A, or P282A mutant WWOX proteins were treated with 20 μM cisplatin for 24 hours, followed by flow cytometric analysis of Annexin V-positive cells. CAL-62 cells stably overexpressing wild-type WWOX exhibit fewer cisplatin-induced apoptotic cells compared to those expressing the WWOX^P252A^ or WWOX^P282A^ mutant. Data are presented as mean ± SEM from three independent experiments.

**Supplementary Figure 8:**


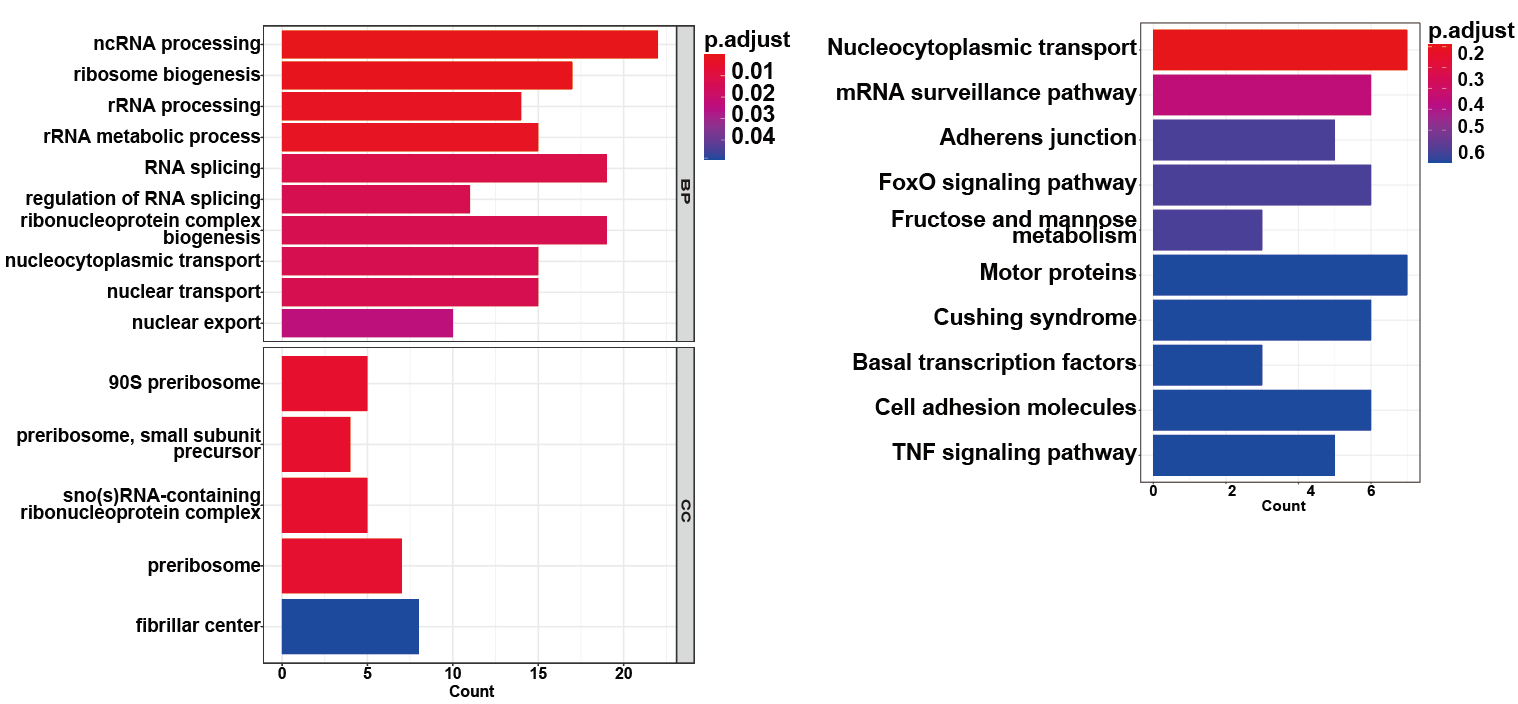


**Supplementary Figure 8. Bar chart of GO and KEGG pathway enrichment analysis on 261 putative WWOX-interacting proteins identified by IP-MS.** Enrichment significance was determined using an adjusted P-value < 0.05. The top 10 most significantly enriched terms are shown.

**Supplementary Figure 9:**


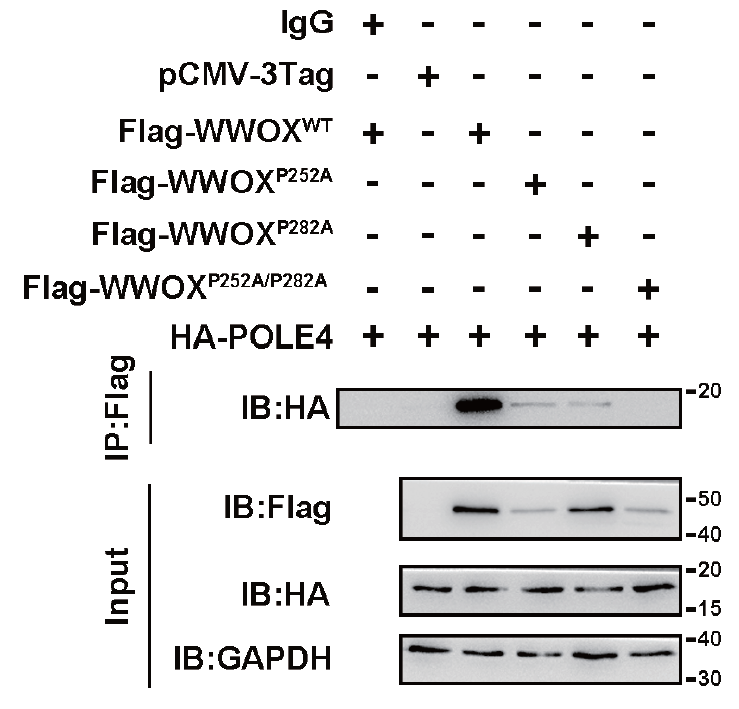


**Supplementary Figure 9. Co-IP shows the interaction of WWOX and POLE4.** HEK293T cells were co-transfected with Flag-tagged vectors expressing either wild-type WWOX (WWOX^WT^) or mutants (WWOX^P252A^, WWOX^P282A^, WWOX^P252A/P282A^), together with a HA-tagged POLE4 vector. After UV exposure, cell lysates were immunoprecipitated with anti-FLAG or IgG control antibody, followed by western blot for POLE4 (HA-tagged).

**Supplementary Figure 10:**


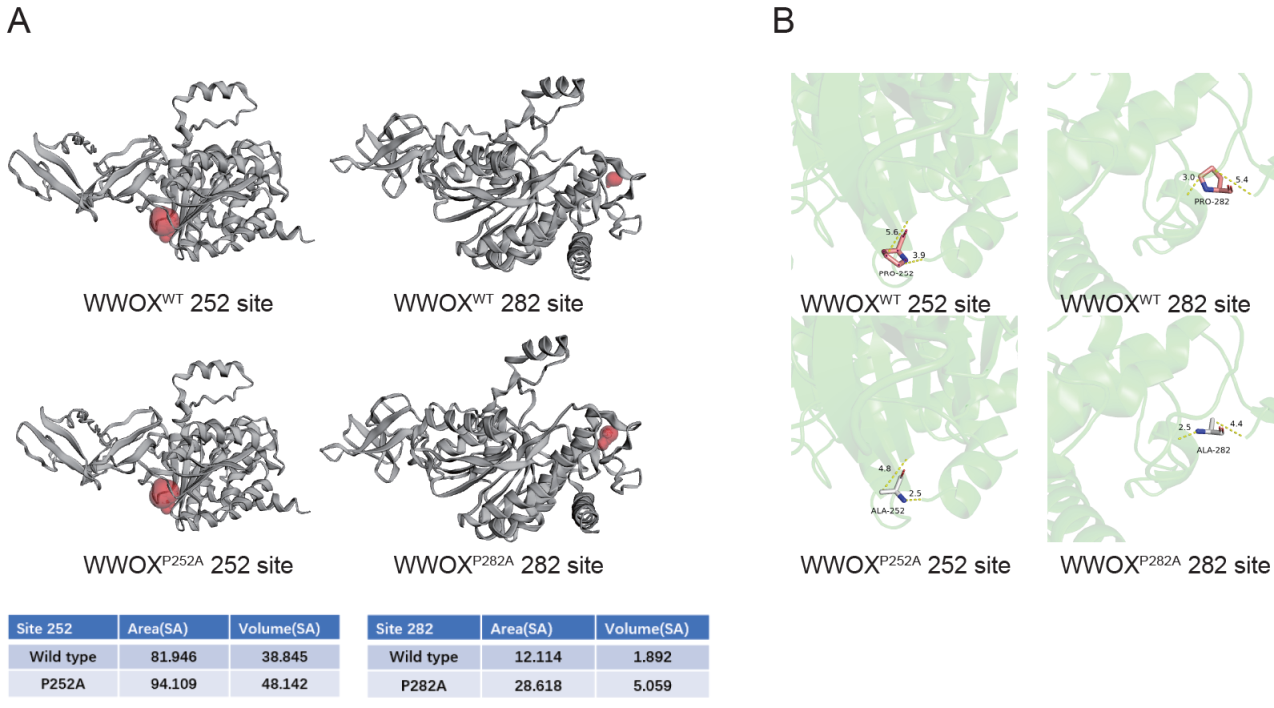


**Supplementary Figure 10. The three-dimensional structures of the wild-type WWOX protein and its P252A and P282A mutants. (A)** Geometric and topological properties of the wild-type and mutant (P252A and P282A) WWOX protein structures at residues 252 and 282 were analyzed using PyMOL and CASTp 3.0 software. The red shaded areas are substrate binding pockets. **(B)** The hydrogen bond formation at residues 252 and 282 of wild-type and mutant WWOX proteins using PyMOL software.

**Supplementary Figure 11:**


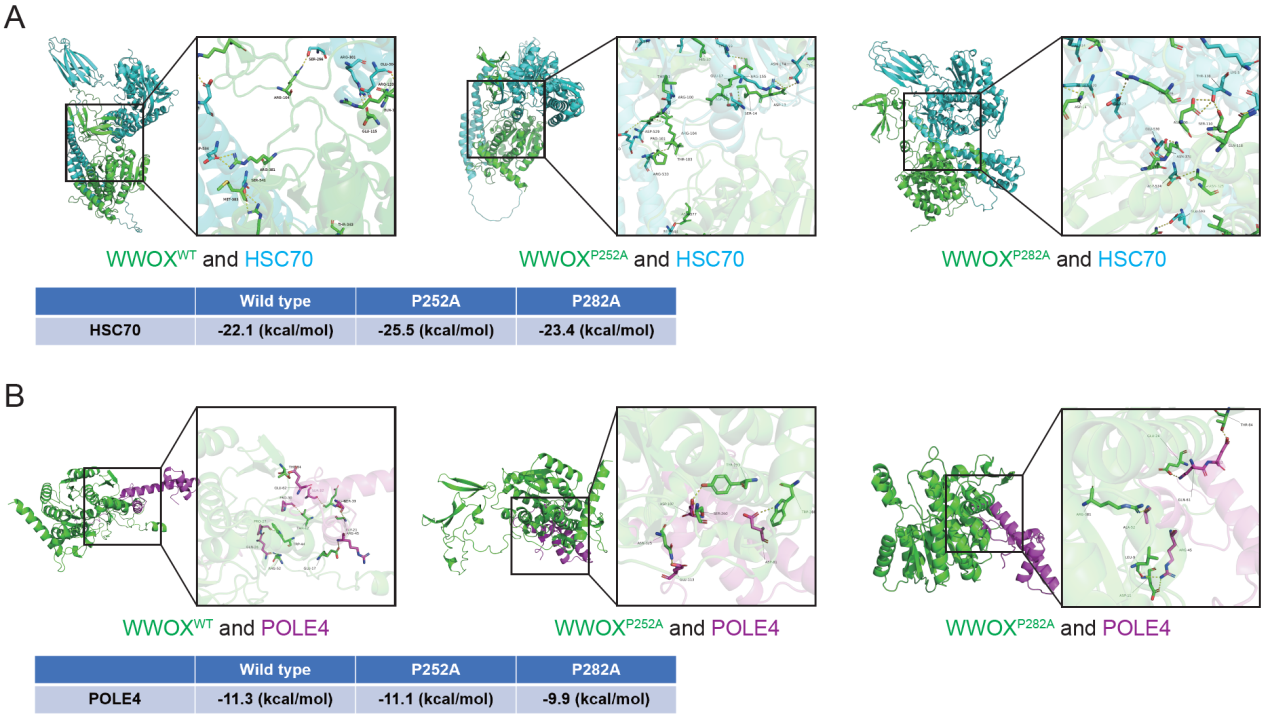


**Supplementary Figure 11. Molecular docking analysis of the WWOX protein (wild-type, P252A and P282A mutants) with its binding partners, HSC70 and POLE4. (A)** Representative structure of molecular docking between WWOX (green) and HSC70 (blue) simulated by alphafold3. The binding energy analyzed by PRODIGY is shown at the bottom. **(B)** Representative structure of molecular docking between WWOX (green) and POLE4 (purple) simulated by alphafold3. The binding energy analyzed by PRODIGY is shown at the bottom.
